# Identifying a potential role of immune cells in gadolinium deposition within the brain

**DOI:** 10.1186/s12987-025-00674-5

**Published:** 2025-07-29

**Authors:** Dixy Parakkattel, Nico Ruprecht, Peter Broekmann, Sarah Guimbal, Chiara Stüdle, Sasha Soldati, Johannes T. Heverhagen, Britta Engelhardt, Hendrik von Tengg-Kobligk

**Affiliations:** 1 Department of Diagnostic, Interventional and Pediatric Radiology, Inselspital, University of Bern, Bern, Switzerland; 2 Department of BioMedical Research, University of Bern, Bern, Switzerland; 3 Department of Chemistry, Biochemistry and Pharmaceutical Sciences, University of Bern, Bern, Switzerland; 4 Theodor Kocher Institute, University of Bern, Bern, Switzerland

**Keywords:** Gadolinium-based contrast agents, Immune cell migration, Blood-brain barrier

## Abstract

**Background:**

Gadolinium (Gd) deposition in the brain was observed in patients with history of gadolinium-based contrast agent (GBCA) administration. However, the exact mechanism behind this deposition remains unclear, especially given that an intact blood-brain barrier (BBB) is considered impermeable to GBCA. In this study, we propose that immune cells might play a role in facilitating GBCA entry into the brain despite an intact BBB.

**Methods:**

Gadoterate meglumine, gadoteridol, gadobutrol and gadodiamide were investigated as GBCAs. Immune cells from human donor buffy coats were isolated, incubated with the GBCA and used in the experiments. Gd associated with the immune cells were measured using single-cell inductively coupled mass spectrometry (SC-ICP-MS). Flow cytometry analysis was performed to characterise the adhesion molecule expression profile on the immune cells and binding assay was employed to check the binding of Gd treated immune cells with endothelial ligands in static conditions. An in vitro model of the human BBB that prevents free diffusion of GBCA across was further used to observe immune cell behaviour at the BBB under physiological flow, in vitro.

**Results:**

Our findings confirm that various immune cells, including CD4^+^ T cells, CD8^+^ T cells, monocytes, NK cells and B cells are capable of taking up the different GBCAs. Furthermore, we demonstrate that GBCA loading does not impair immune cell interaction with the endothelial ligands required for successful extravasation across the BBB under static conditions. Most importantly, we show that T cells and monocytes, loaded with the different contrast agents, extravasated across an in vitro BBB model under physiological flow conditions in a comparable manner to non GBCA loaded cells.

**Conclusions:**

Taken together, our in vitro observations show that immune cells can transport GBCA across the BBB and could lead to permanent deposition of Gd in the brain.

**Supplementary Information:**

The online version contains supplementary material available at 10.1186/s12987-025-00674-5.

## Introduction

Gadolinium-based contrast agents (GBCAs) are employed in magnetic resonance imaging (MRI) to improve contrast between tissues and thereby improve the diagnostic value of MRI examinations in treating a wide variety of diseases including malignancies, inflammation, and degenerative, hereditary and vascular diseases [1]. Since GBCA introduction to the market in the late 1980s, millions of doses have been administered and well tolerated by patients worldwide [2, 3]. However, it was shown in 2006 that frequent and/or high dose application of contrast agents in patients with severe renal insufficiency can lead to the development of a rare systemic disease called nephrogenic systemic fibrosis (NSF) [4]. It has been demonstrated that the deposition of gadolinium (Gd) in various tissues and organs is responsible for the clinical symptoms of this potentially life-threatening disease [5] though the exact pathogenesis is yet to be clearly understood. Following a first report in 2014 showing presence of Gd in brain in patients with GBCA administration history, even with intact renal function, several other groups worldwide have confirmed brain Gd deposition in both human and animal model studies [2, 3, 6]. Following a major response from the scientific community, regulatory authorities and radiological societies, who issued warnings, guidelines and recommendations on the use of GBCAs in daily medical practice and in the treatment of patients with renal insufficiency, the number of new NSF cases has decreased significantly [5]. Initially, it was hypothesized that Gd deposition in the brain occurred in patients with conditions that could lead to blood-brain barrier (BBB) abnormalities. However, subsequent reports have confirmed the presence of brain Gd deposition even in patients with presumably an intact BBB [7].

The BBB is one of the central nervous system (CNS) barriers formed by specialized brain microvascular endothelial cells with low pinocytic activity and with continuous and complex tight junctions present between the cells [8, 9]. The BBB establishes a highly selective barrier, regulating the bidirectional transport of substances between the peripheral blood and the brain parenchyma [8]. In addition, the BBB strictly regulates immune cell trafficking into the CNS, allowing select immune cells to traverse across to perform CNS immune surveillance of the otherwise immune privileged CNS environment [10]. In particular, peripherally activated T cells cross the BBB even in the absence of neuroinflammation, albeit in low numbers [8]. Other cell types, such as B cells, monocytes and neutrophils are also studied in this regard [10]. This process has been extensively investigated in peripherally activated T cells, B cells and monocytes due to their involvement in possible induction and/or progress of neurological disorders such as multiple sclerosis [11–13]. Immune cell extravasation across the BBB is facilitated by highly orchestrated sequential interactions between immune cell adhesion molecules, including P-selectin glycoprotein-1 (PSGL-1), α4β1-integrin and αLβ2-integrin, and endothelial ligands such as P-selectin, vascular cell adhesion molecules-1 (VCAM-1) and intercellular adhesion molecule-1 (ICAM-1) respectively as described previously [14].

Building on our previous findings of GBCA uptake in immune cells in patients undergoing contrast enhanced MRI [15], we here aimed to investigate if the immune cells that uptake GBCA can cross the BBB with GBCA as a load and thereby facilitate GBCA access into the brain. Here, we employed an in vitro BBB model with human brain microvascular endothelial cell-like cells (BMEC-like cells) to study the immune cell behaviour at the BBB post GBCA loading [16]. BMEC-like cells are differentiated from human induced pluripotent stem cells (hiPSCs) by the extended endothelial cell culture method (EECM) [16]. EECM-BMEC-like cells show barrier properties (transendothelial electrical resistance, (TEER) [17] and small molecule permeability [18]) comparable to primary human BMECs with junctional localisation of the tight junction proteins claudin-5 and occludin, the adherens junction protein VE-cadherin and the junctional scaffolding protein zona occludens-1 (ZO-1). Moreover, exhibiting a mature immune phenotype resembling that of primary human BMECs with expression of key adhesion molecules including ICAM-1, ICAM-2 and VCAM-1, this model is an excellent choice to investigate immune cell interactions with the BBB which happens at the post-capillary venules [16].

Here, we quantified the GBCA loading in different immune cell subsets from the innate immune system, namely monocytes, neutrophils and NK cells as well as by CD4^+^ T cells, CD8^+^ T cells and B cells from the adaptive immune system. In this study, we focussed on four different GBCAs- gadoteridol, gadoterate dimeglumine, gadodiamide and gadobuterol. Gadodiamide is a linear agent with a flexible ligand, while others are macrocyclic with rigid caged structures. Previous research has shown that GBCAs differ in their stability with macrocyclic agents being more stable in retaining the Gd^3+^ ion compared to linear agents [19]. Consequently, in our study, we included the three available macrocyclic agents that are in the market and included the linear agent to which most of the NSF cases and Gd deposition were attributed to [20]. GBCA loading did not affect the cell surface expression of adhesion molecules on the different immune cell subsets nor their ability to bind to purified endothelial adhesion molecules under static conditions. Finally, we demonstrate that GBCA loading did not interfere with immune cell migration across the BBB under physiological flow in vitro.

Taken together our data show that different circulating immune cell subsets can take up GBCA. While adaptive immune cells may thus carry GBCA across the BBB even in the absence of neuroinflammation, innate immune cells may also cross the inflamed BBB. In both cases, CNS infiltrating immune cells may contribute to GBCA deposition in the brain.

## Materials and methods

### PBMC isolation and further subpopulation isolations

Peripheral blood mononuclear cells (PBMCs) were isolated from buffy coats purchased from Swiss Red Cross (Interregionale Blutspende SRK, Bern, Switzerland, project no. P_172) using Ficoll-Paque Plus (Cytiva) density gradient. The buffy coats were obtained from the blood bank, therefore no information with respect to age or sex of the donors is available. All donors are presumed healthy as they must meet the criteria to donate blood. Isolated PBMCs were stored in vapour phase in liquid nitrogen tank until use. The buffy coats used in each experiment is provided in Table S4. Further isolation of CD4^+^ T cells (Catalog #17952), CD8^+^ T cells (Catalog #17953), monocytes (Catalog #19359), NK cells (Catalog #17955) and B cells (Catalog #17954) from PBMCs was performed employing immunomagnetic negative selection isolation with EasySep kits from StemCell Technologies. Neutrophils were isolated from the granulocyte-erythrocyte fraction after Ficoll-Paque Plus density gradient centrifugation. Initially, the erythrocytes were lysed using ACK lysing buffer composed of 155 mM ammonium chloride, 10 mM potassium bicarbonate and 0.10 mM disodium EDTA in distilled water and from the remaining granulocytes, neutrophils were isolated employing immunomagnetic negative selection isolation with EasySep kit from StemCell Technologies (Catalog #17957). The purity of each of the isolated cells is shown in Fig. S4.

### PBMC culture media

PBMCs were stored in culture media with RPMI 1640 containing 1% L-glutamine, 1% sodium pyruvate, 1% nonessential amino acids, 1% penicillin/streptomycin, 1% kanamycin, 10% fetal bovine serum (FBS, Hyclone), 0.00005% b-MeEtOH and 10U/ml IL-2 until experiments.

### In vitro activation of CD4^+^ and CD8^+^ T cells

Isolated CD4^+^ T cells and CD8^+^ T cells were activated in 48 well plate at 0.5 million cells/mL. Activation of CD4^+^ and CD8^+^ T cells were performed with agonistic antibodies against CD3 (Catalog no.: 317326, Biolegend) and CD28 (Catalog no.: 302902, Biolegend) over a 5-day period in PBMC culture media at 37 °C, 5% CO_2_.

### Culture of monocytes

The isolated cells were cultured in RPMI 1640 containing 10% FBS, 1% penicillin/streptomycin, 2% L-glutamine until experiments [21].

### Culture of B cells and NK cells for binding assay

B cells and NK cells were cultured in RPMI 1640 containing 10% FBS (Hyclone) until experiments.

### GBCA loading studies

In the clinics, GBCAs are administered at around 2 mM in blood and rapidly cleared via the kidneys with elimination of 75% of the administered dose within the first 4 h in a recipient with normal renal function (eGFR > 60 mL/min/1.73 m^2^) [22]. Therefore, the initial 2 mM GBCA concentration will not be maintained in the blood for more than a few hours. Based on these considerations, we performed our experiments with incubations at 2 mM final concentration for one hour at 37 °C. Cells at 1 × 10^6^ cells/mL were incubated with GBCA to a final concentration of 2 mM for 1 h at 37 °C, 5% CO_2_. Commercial GBCAs used in the study, gadoterate meglumine (Dotarem, Guerbet, France), gadoteridol (ProHance, Bracco, Italy), gadobutrol (Gadovist, Bayer Healthcare, Germany) and gadodiamide (Omniscan, GE Healthcare, USA), were obtained from Insel Hospital Pharmacy (Bern, Switzerland). The cells were then washed once and fixed with 1X IC fixation buffer (eBioscience Thermo Fischer Scientific, Waltham, MA, USA) for 30 min at room temperature. Subsequently, cells were washed twice with PBS with 0.5% BSA and finally resuspended in dPBS to a final concentration of 0.5 × 10^6^ cells/mL. Cellular Gd measurements were performed using NexION 2000 ICP-MS (Perkin Elmer, Waltham, MA, USA) as described previously [23]. The cell samples were loaded at a concentration of 100k cells/mL, diluted with PBS and the results obtained were analysed using Syngistix Single Cell Application Software version 3.5 (PerkinElmer, Waltham, MA, USA). Performance check was performed prior to use followed by calculating transport efficiency of the system using 50 nm gold nanoparticles (NanoComposix, San Diego, CA, USA). SC-ICP-MS was calibrated for Mg and Gd by measuring stock solutions with defined Mg and Gd concentrations of 1, 5, 10 and 20 ppb in 2% HNO_3_ producing a calibration curve which was further employed for measuring the elemental concentration in the later introduced samples. To ensure the sample introduction as single cells, the cells were syringed once using a 25-guage syringe needle.

### Flow cytometry

The adhesion molecule expression profile analysis of PBMC subpopulations – CD4^+^ T cells, CD8^+^ T cells, B cells, monocytes and NK cells was performed using flow cytometry. The different adhesion molecules (α4-, β1-, αL-, β7- and PSGL-1) and the subpopulation markers were targeted using fluorochrome conjugated antibodies on live cells. Detailed information about the employed antibodies and isotype controls, and the targeted subpopulation markers can be found in Table S1 and Table S2. 1 × 10^6^ PBMCs were incubated with GBCA at a final concentration of 2 mM at 37 °C, 5% CO_2_ for 1 h. Cells without any treatment were used as control. Subsequently, 2 × 10^5^ cells were transferred to 96-well microtiter plate, washed once in the wash buffer followed by media change to FACS buffer (2.5% (v/v) heat inactivated FBS, 0.1% (w/v) Sodium Azide (Sigma-Aldrich) in dPBS (Gibco)) and then incubated with a cocktail of fluorochrome conjugated antibodies for 30 min at 4 °C. Post incubation, the cells were washed twice with FACS buffer and the cell surface adhesion molecule expression was measured with Attune NxT Flow Cytometer (Thermofisher Scientific, Switzerland) using Attune NxT Software v3.1.2. Data analysis was performed using FlowJoTM 10 software (Tree Star, Ashland, OR, USA). The gating strategy used for different subpopulations is given in Fig. S2a.

The cell surface adhesion molecule expression profile analysis of in vitro activated CD4^+^ T cells and CD8^+^ T cells, activated in presence of agonistic antibodies against CD3 and CD28 over a 5-day period in PBMC culture media (as described before) was performed (Fig. S3a). 1 × 10^6^ cells were incubated with GBCA at a final concentration of 2 mM at 37 °C, 5% CO_2_ for 1 h. Cells without any treatment were used as control. The different adhesion molecules (α4-, β1-, αL-, β7- and PSGL-1) were targeted using fluorochrome conjugated antibodies on live cells (Table S1). Further details are the same as with PBMCs as discussed before.

### Binding assays

The binding assays of PBMCs and the different subpopulations to human BBB recombinant ICAM-1 or VCAM-1 were performed. In brief, wells of 12-well (5.2 mm) slides (Epredia speciality printed microscope slide, Ref XEXER202W) were coated directly with either 10 µg/mL human recombinant ICAM-1/CD54 Fc chimera protein (Catalog no.: 720-IC-050, R&D systems) or human recombinant VCAM-1 Fc chimera protein (Catalog no.: 553706, BioLegend) in PBS for 2 h at 37 °C, 5% CO_2_. The wells were then blocked with 1.5% (v/v) bovine albumin serum in dPBS overnight at 4 °C. Human recombinant Delta/Notch-like EGF-related receptor (DNER, 3646-DN-050, R&D systems) was employed as negative control to test for unspecific cell-to-slide interactions.

Immune cells, untreated or incubated with GBCA for 1 h at 37 °C, 5% CO_2_, were added to the wells and allowed to bind for 30 min at room temperature on a levelled rotating platform to ensure even distribution of cells in the well. The wells were washed twice in PBS to remove unbound cells, followed by 2-hour fixation of the ligand bound cells in 2.5% glutaraldehyde in PBS on ice. Later, the slides were washed twice in PBS and mounted using mowiol. Images of wells were acquired using an epifluorescence microscope Nikon Eclipse Ni-U. Counting of bound cells were automated employing Image J software version 2.14.0 (NIH, Bethesda, MD, USA). During the study period, there was a change of the camera to a Nikon Digital Sight 10 (DS-10) camera with Nikon NIS-Elements BR 5.42.06 software (Nikon, Egg, Switzerland) changing the field of view over which the images were acquired. Therefore, the images captured after the change were cropped in Fiji to the area as the older images and cell counting performed on the cropped images as stated previously.

### In vitro BBB model – EECM-BMEC like cells

Extended endothelial cell culture method (EECM)- differentiated brain microvascular endothelial cell (BMEC)-like cells derived from human induced pluripotent stem cells (hiPSCs) [24] from healthy donors (cell line ID: LNISi001-A, LNISi002-A and LNISi002-B) were described before [16, 25]. The smooth muscle-like cells (SMLC) that arise during the differentiation were separated and cultured to collect SMLC-condition media exactly as described [25]. EECM-BMEC-like cells at passages 4–6 were cultured to confluency on collagen IV coated 6 well plates prior to use in the experiments as these were shown to exhibit good barrier properties against NaFl [16].

### Permeability assay

EECM-BMEC-like cells were used to form the in vitro BBB model as described [25]. Briefly, on Day 0, confluently grown EECM-BMEC-like cells were added to Collagen IV/ Fibronectin/ water coated 0.4 μm pore size Transwell filter inserts (PC membrane, 0.4 μm, 12 mm, Cat#3401, Corning). With media changed on the top and bottom chambers of the filter every 2 days, the cells formed a monolayer on Day 6 and the assay was performed as described previously [25]. Briefly, sodium fluorescein (NaFl, 376.3 Da, Sigma-Aldrich) at 10 µM final concentration and GBCA at 2 mM final concentration were added to the top chamber of the filters and samples collected from the bottom chamber every 15 min up to one hour and from the top chamber at the one-hour time point. An empty non-coated filter was used as positive control. The collected samples were analysed for NaFl presence using Tecan Infinite M1000 multi-well plate reader (Tecan Trading AG) and Gd measurements using NexION 2000 ICP-MS (Perkin Elmer, Waltham, MA, USA). The NaFl permeability coefficient was calculated as described previously [26]. For Gd measurements, the system was calibrated using Gd standards of defined concentrations to create a calibration curve. The samples were then diluted as: samples from Transwell filter bottom chamber and top chamber were diluted 100 and 4000/5000 times respectively in 2% HNO_3_ (to ensure measured Gd values were within the calibration curve). The Gd concentration in original samples were calculated from the measured Gd values according to the initial dilution performed. Europium was used as an internal control for ICP-MS measurements. Permeability coefficient of Gd across the BBB in vitro model was calculated similarly as NaFl permeability coefficient.

### Immunofluorescence staining

EECM-BMEC-like cells were cultured on Transwell filters as described under permeability assay in Materials and Methods and stained as described [27]. To stain for claudin-5, VE-cadherin and ZO-1, the cells on the filters were fixed with − 20 °C MeOH followed by blocking and permeabilization with 5% skimmed milk with 0.1% Triton X-100. Primary antibody staining was performed for 1 h at room temperature, followed by one hour of secondary antibody staining at room temperature. The filters were then mounted with mowiol and images acquired with Nikon Eclipse Ni-U microscope connected to a Nikon DS-10 camera with Nikon NIS-Elements BR 5.42.06 software (Nikon, Egg, Switzerland). Information about the antibodies used listed in Table S3.

### In vitro live cell imaging

In vitro live cell imaging to investigate the immune cell behaviour at the BBB under physiological flow was performed as described previously [27, 28]. Briefly, EECM-BMEC-like cells were grown to confluency within a silicone cloning ring placed on collagen IV/fibronectin coated µ-Dish (35 mm, low, iBidi). The confluent cells were stimulated with 0.1 ng/ml recombinant human TNFα (R&D systems, Cat#210-TA-020) and recombinant human IFNγ (R&D systems, Cat #285-IF-100) diluted in conditioned media (CM) collected from SMLC from same donor, for 16–24 h at 37 °C, 5% CO_2_. Preparation of SMLC CM is described here [25]. SMLC CM was utilised as it has previously been shown that co-culture of EECM-BMEC-like cells with SMLCs or culture of EECM-BMEC-like cells in the presence of CM from SMLCs stabilises adhesion molecule expression of EECM-BMEC-like cells [16, 25]. Immune cells at 1 million cells/mL were preincubated with GBCA for 1 h at 37 °C in final GBCA concentration of 2 mM and superfused onto the stimulated EECM-BMEC-like cell layer. Before live-cell imaging experiments, immune cells were changed into migration assay medium (MAM) of DMEM without phenol red containing HEPES (25 mM), 5% FBS (Hyclone) and 2 mM L-glutamine and the system was in the MAM medium throughout the experiment. Untreated immune cells were used as control. 

The low shear of 0.1 dyne/cm^2^ was applied for the first 5 min and immune cells were allowed to accumulate on EECM-BMEC-like cell monolayer within the flow chamber for 4 min (accumulation phase). The shear was then increased to 1.5 dyne/cm^2^, thereby increasing the shear in the system to physiological shear (shear phase). The physiological shear was then applied for 20 min i.e., until the end of the experiment. Thirty seconds after induction of the shear phase, the number of cells arrested on to the cell layer was quantified as the total no. of cells arrested and the post arrest behaviour of the arrested cells was assessed during the shear phase. Images were obtained with phase-contrast at 10x magnification every 5–10 s, to create time-lapse videos, with an inverted microscope (AxioObserver, Carl Zeiss, Oberkochen, Germany) using a Zeiss Axiocam MRm camera and Zen Blue 3.3 version software. The exhibited behaviours of immune cells were manually analysed frame-by-frame of the time-lapse videos using ImageJ software (ImageJ software, National Institute of Health, Bethesda, USA) and were categorised as described previously [29]. In brief, immune cells that remained bound to the monolayer 30 s after the onset of physiological shear onset were counted as ‘arrested cells’. The total no. of arrested cells was set to 100% and the different behaviours of the arrested cells as described were expressed as fractions thereof. From the arrested cells, immune cells that polarised and crawled over the monolayer during the observation time were categorised as ‘crawling’. However, if after polarisation, the displacement of the cell is less than its own diameter with cell presenting dynamic cellular protrusions, the cell is then referred to as ‘probing’. If the cell migrates across the monolayer after crawling or probing, it is categorised as ‘crawling + diapedesis’ or ‘probing + diapedesis’ respectively. ‘Stationary’ are cells that does not polarise or crawl. Cells that detach from the observational field during the analysis are referred to as have ‘detached’.

### Statistical analysis

Data are shown as the mean ± SD. Comparison between multiple groups was assessed by one-way ANOVA repeated measures or by mixed model (in case of missing values), followed by Tukey’s multiple comparisons test. Ordinary one-way ANOVA was used in analysis of Fig. 4. Paired T-test was performed once for analysis shown in Fig. S5b. Precise statistical analysis and p-value threshold for statistical significance are indicated in the figure legends. Statistical analyses were done using GraphPad Prism 10.4.1 software (Graphpad software, La Jolla, CA, USA).

## Results

### GBCAs are taken up by circulating immune cells

In our previous study, we observed GBCA uptake by immune cells of individuals who underwent contrast enhanced MRI examinations after gadoterate meglumine administration [15]. However, it has remained unclear if all immune cell populations can take up GBCA. To investigate if immune cell subsets from the innate and adaptive immune system can take up GBCAs and if the loading differs between these different immune cell populations or between the different contrast agents, we incubated different immune cell subsets such as CD4^+^ T cells, CD8^+^ T cells, B cells, monocytes and NK cells isolated from healthy donor buffy coats using Ficoll-Paque Plus density gradient with different GBCAs – gadobutrol, gadoterate meglumine, gadoteridol and gadodiamide. The presence of Gd at the cellular level was then measured using single cell inductively coupled mass spectrometry (SC-ICP-MS) as described in the Materials and Methods section.

It has been shown that white blood cell (WBC) incubation for up to 3 h with both linear and macrocyclic GBCAs (gadodiamide and gadoteridol respectively) resulted in recovery of intact chelates from the WBCs [30]. Therefore, we consider the Gd measured from the cells in our experiments to be part of the intact chelate and going forward at instances when elemental gadolinium is measured, we would refer to this as Gd and in regard to cell loading, GBCA.

**Fig. 1:**
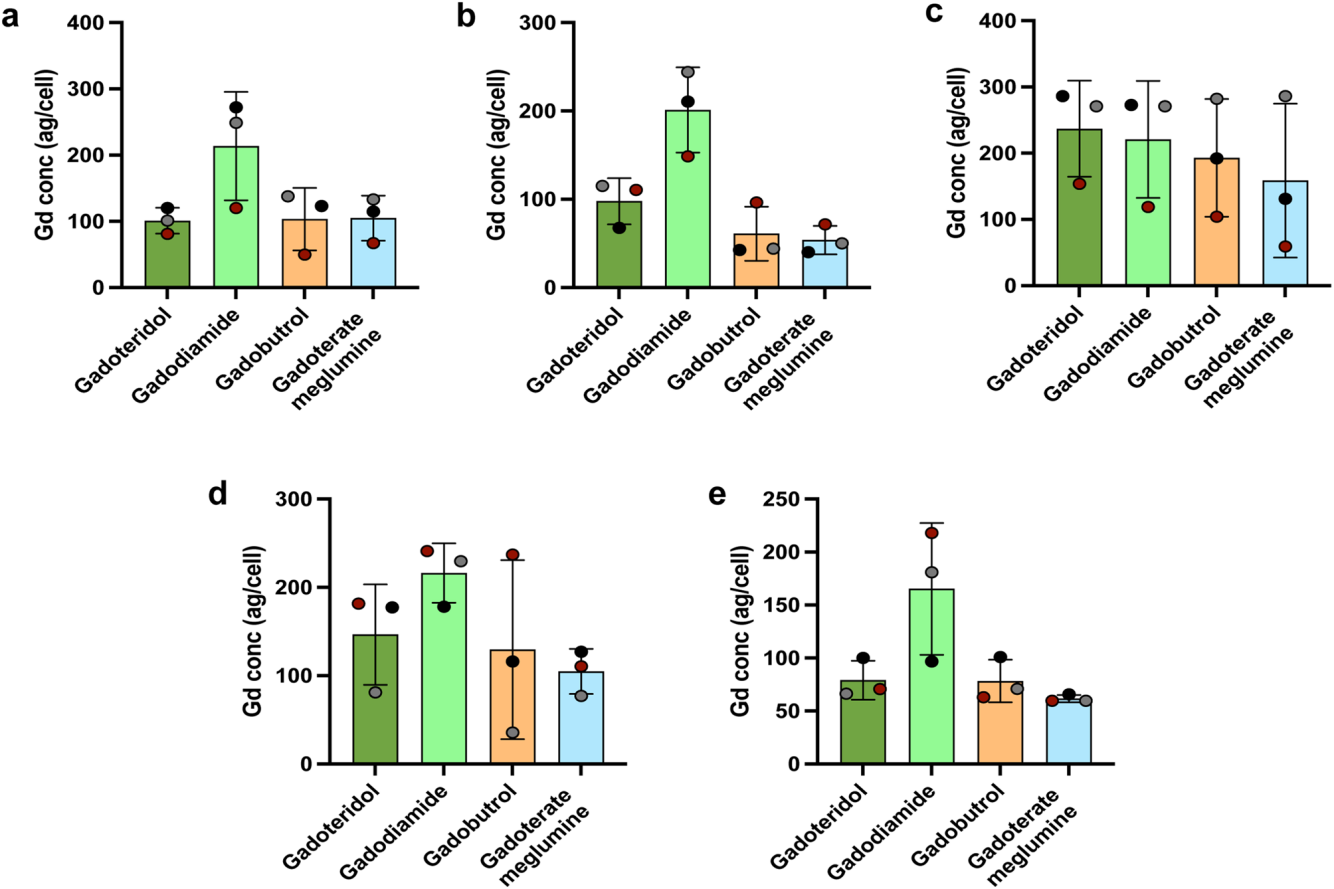
GBCA loading confirmed in circulating immune cells. Gd concentrations measured in circulating **(a)** CD4^+^ T cells, **(b)** CD8^+^ T cells, **(c)** monocytes, **(d)** NK cells and **(e)** B cells are shown. Isolated immune cells at 1 million cells/mL were incubated at a 2 mM final concentration of GBCA for 1 h at 37 °C, 5% CO_2_. Gd concentration in cells was measured using SC-ICP-MS and is shown in attograms/cell (ag/cell). Each dot is the mean value of 1–3 technical replicates, and each colour represents values from a single donor performed as an independent experiment. The donor cells used for the three independent experiments vary between the graphs. Analysis was performed using one-way ANOVA with repeated measures followed by Tukey’s multiple comparisons test and shown as mean ± SD. Further data showing results for in vitro activated CD4^+^ T cells, in vitro activated CD8^+^ T cells and neutrophils are shown in Fig. S1

Comparing the loading of different GBCAs by the different cell types (Fig. 1), Gadodiamide was seen to be taken up to the highest extent by all cells except for monocytes and monocytes showed the highest loading of all the tested GBCAs. Additionally, we also confirmed loading of GBCA in in vitro activated CD4^+^ T cells, CD8^+^ T cells and neutrophils (Fig. S1). Our data thus confirm that GBCA can be taken up by all circulating immune cells.

### Loading of GBCAs does not affect cell surface expression of adhesion molecules on circulating immune cells nor their ability to interact with ICAM-1 and VCAM-1

Leukocyte adhesion molecules such as PSGL-1, α4β1-integrin (VLA-4) and αLβ2-integrin (LFA-1) are instrumental for immune cell migration across the BBB during CNS immune surveillance and neuroinflammation [10]. Therefore, we next investigated whether the loading of GBCA would affect the cell surface expression of these molecules on different immune cell subsets. To this end, we incubated PBMCs isolated from donor buffy coats with the four different contrast agents and performed multicolour flow cytometry for CD4^+^ T cells, CD8^+^ T cells, CD14^+^ monocytes, CD16^+^ 56^+^ NK cells and CD19^+^ B cells as well as for cell surface expression of PSGL-1 and the α4-, β1-, αL-, β2- integrin subunits.

**Fig. 2:**
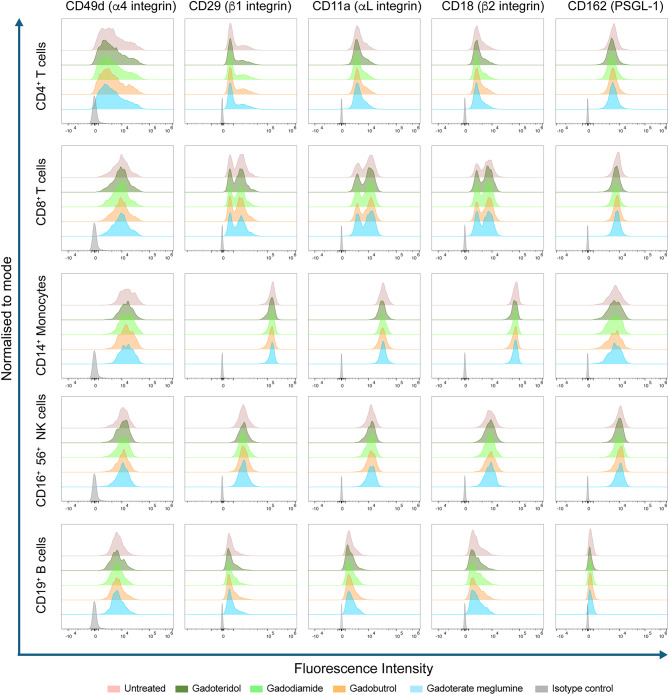
Cell surface adhesion molecule expression on immune cells is unaltered after GBCA treatment of the cells. PBMCs at 1 million cells/mL were incubated at a 2 mM final concentration of GBCA for 1 h at 37 °C, 5% CO_2_. Cells incubated with cell culture media only was used as control. Flow cytometry analysis for α4, αL, β1, β2 and PSGL-1 cell surface expression on CD4^+^ T cells, CD8^+^ T cells, monocytes, NK cells and B cells within PBMCs are shown. Each colour represents treatment with a different GBCA. Cells incubated with cell culture media only (Untreated) was used as control and isotype control is shown in grey. Plots are representative of 4 independent experiments with cells from 4 different donors. The gating strategy for each immune cell subset shown in Fig. S2(**a**). Further quantifications are shown in Fig. S2(**b-d**). The panel of fluorescently labelled antibodies used is provided in Table S1

The results confirmed expression of all the tested adhesion molecules on the different immune cell subpopulations targeted (Fig. 2). Analysing the results and comparing the percentage of cells expressing high or low levels of α4 integrins in CD4^+^ T cells and CD8^+^ T cells and total α4 expression in monocytes, NK cells and B cells, we observed no significant difference in the cell surface expression of α4 integrins in any of the immune cell populations studied irrespective of GBCA loading or the type of GBCA studied (Fig. S2b-i). This was true for all the studied integrins. Collectively, our data show that GBCA loading did not impact cell surface expression of adhesion molecules on circulating immune cells (Fig. 2).

**Fig. 3:**
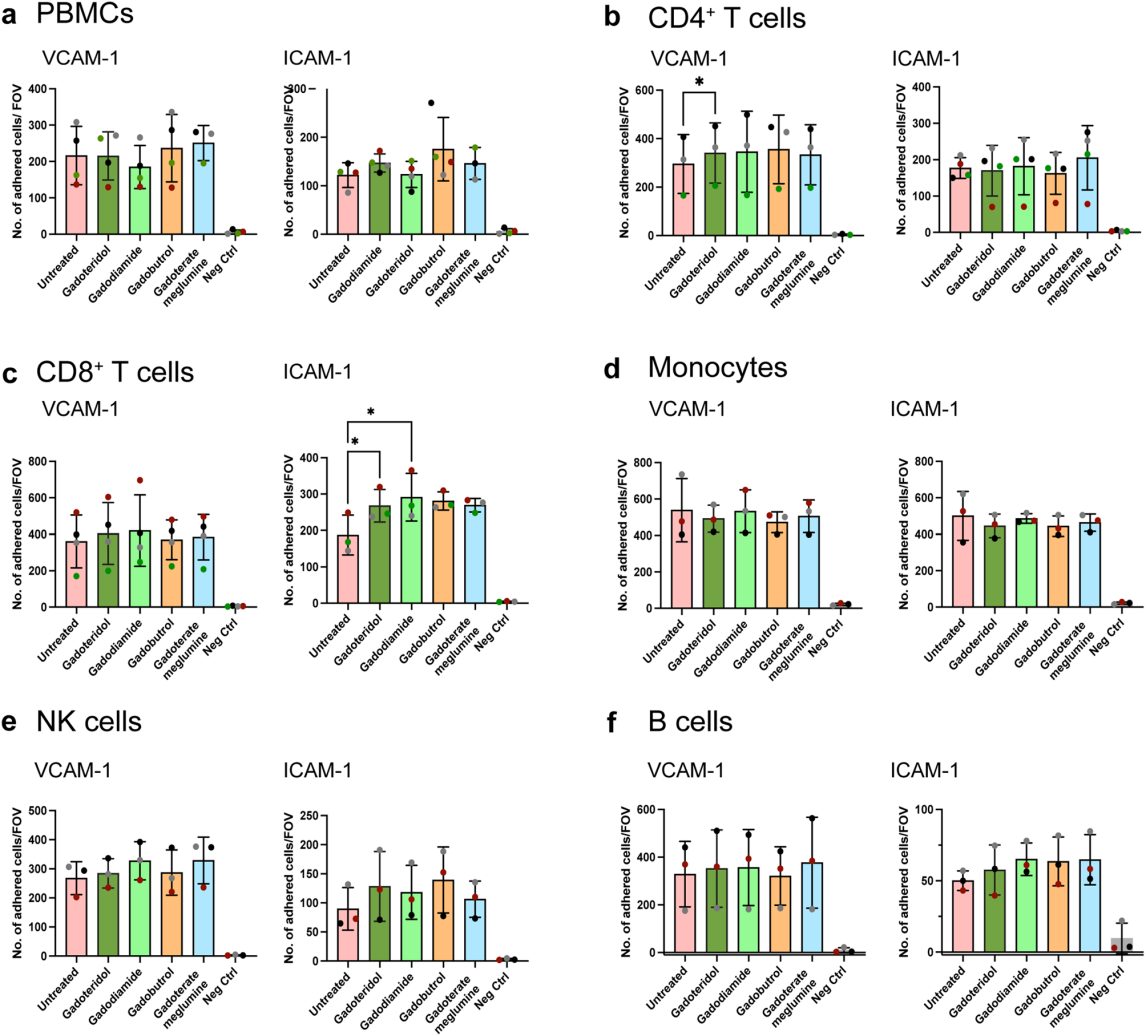
GBCA loading does not affect immune cell binding to immobilised recombinant VCAM-1 or ICAM-1 in static conditions. Isolated immune cells at 1 million cells/mL were incubated at a 2 mM final GBCA concentration for 1 h at 37 °C, 5% CO_2_. Number of **(a)** PBMCs, **(b)** CD4^+^ T cells, **(c)** CD8^+^ T cells, **(d)** monocytes, **(e)** NK cells and **(f)** B cells adhering to immobilised human recombinant VCAM-1 or ICAM-1 under static conditions are shown. Cells incubated with cell culture media only (Untreated) was used as control. Each dot is the mean value of 2–3 technical replicate measurements, and each colour represents values from a single donor performed as an independent experiment. The donor cells used for the three independent experiments varies between the graphs. Immobilised human recombinant DNER was used as negative binding control (Neg Ctrl). Analysis performed using One-way ANOVA with repeated measures or mixed-effect model if values missing (in Fig. 4 (a) ICAM-1) and followed by Tukey’s multiple comparisons test and shown as mean ± SD (*p* < 0.05=*)

Although we found that cell surface expression of α4-, β1-, αL-, β2- integrins and PSGL-1 were unaltered on circulating immune cells post GBCA loading, this did not allow to conclude that GBCA would not affect the function of the respective adhesion molecules. Especially, integrins are constitutively expressed on circulating immune cells but obtain their functional activity by conformational changes to a high affinity conformation by inside-out-activation of the integrins which is crucial for the extravasation process [31]. Therefore, we next asked if the respective adhesion molecules maintained their affinity to endothelial ligands on GBCA treated immune cells. To this end we compared the binding of PBMCs (Fig. 3a), and subsequently the different immune subpopulations (Fig. 3b-f), incubated or not with the different GBCAs to immobilised human recombinant ICAM-1 or VCAM-1. Comparing with the number of bound untreated PBMCs, GBCA loading did not show any statistically significant difference on the interaction of PBMCs with either ICAM-1 or VCAM-1. This was also true for all the subsets studied except for CD4^+^ T cells that showed significantly higher number of gadoteridol treated cells binding to VCAM-1 compared to untreated cells and CD8^+^ T cells that showed significantly higher numbers of gadoteridol and gadodiamide treated cells binding to ICAM-1 compared to untreated cells (Fig. 3c). Taken together, these data show that GBCA loading did not impair functional cell surface expression of α4β1-integrins and αLβ2-integrins on T cells, B cells, monocytes and NK cells.

### A human in vitro BBB model prohibiting passage of GBCAs

Under steady state conditions, the barrier properties of the BBB prohibit free passage of circulating GBCA into the CNS [32]. Therefore, GBCAs are routinely used in the clinics to detect BBB disruptions in assessment of various inflammatory, and infectious diseases [33]. Specifically, GBCA enhancing lesions are an early pathological hallmark and thus a diagnostic criterium in multiple sclerosis [34].

To test if GBCA loaded immune cells can cross the BBB, and potentially carry GBCAs across this barrier, we had to identify an in vitro model of the human BBB that does not allow for free diffusion of GBCA. To this end, we made use of our recently established in vitro BBB model with BMEC-like cells differentiated from hiPSCs by EECM [16, 25]. These EECM-BMEC-like cells have been shown to establish barrier properties comparable to primary human BMECs and importantly also display a mature immune phenotype allowing to study immune cell interactions with the BBB in vitro [16].

**Fig. 4:**
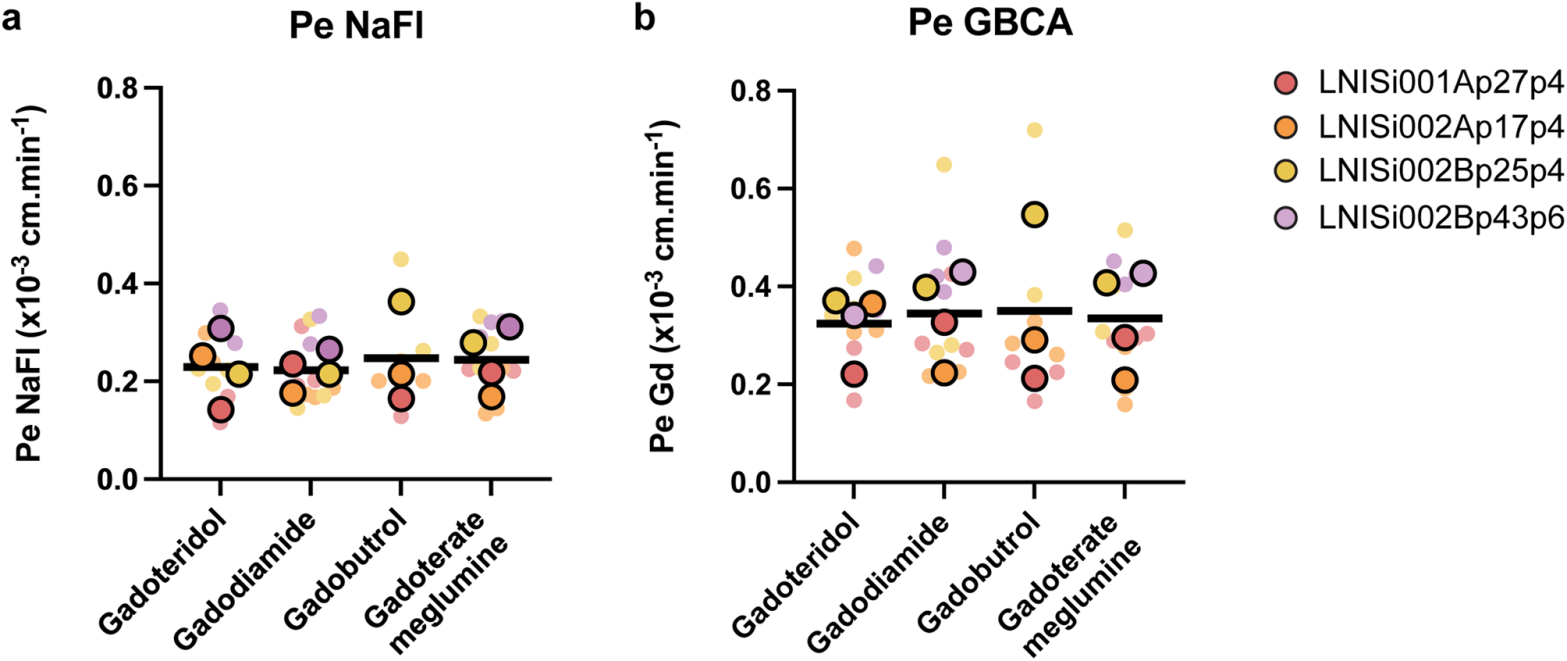
GBCAs do not diffuse across EECM-BMEC-like cells. Permeability coefficient (Pe) values of NaFl and GBCA (measured as Gd) across the EECM-BMEC-like cell in vitro BBB model at 37 °C, 5% CO_2_ measured over an hour is shown. EECM-BMEC-like cells were cultured to confluency on 0.4 mm pore size Transwell filters for 6 days and permeability was measured on Day 6. To each filter, NaFl 10 mM and a GBCA (2 mM final concentration) was added and NaFl values and Gd measurements were performed from the same collected samples. The sampling strategy and Pe calculation are described in Materials and Methods. **(a)** Pe calculated for NaFl and **(b)** for different GBCAs across the EECM-BMEC-like cells from four independent experiments with each independent experiment performed using cells from a different donor or passage. Small dots of each colour show Pe from a single filter, and the bigger dot of the same colour is the mean Pe of NaFl/GBCA from the experiment. The mean Pe from each independent experiment was then used to calculate the Pe value as mean ± SD for NaFl/GBCA (given in Results section). Ordinary one-way ANOVA followed by Tukey’s multiple comparisons test was used to compare results of Pe NaFl/Pe Gd obtained for each GBCA

Our previous studies showed that EECM-BMEC-like cells show low permeability to the small fluorescent tracer sodium fluorescein (NaFl, 376.3 Da) [16, 27]. From an initial experiment comparing the permeability of gadoterate meglumine (0.754 kDa) and NaFl across EECM-BMEC-like cells, individually or in combination, showed that there was no significant difference between the Pe measured either for NaFl or the GBCA (measured as Gd by ICP-MS) when studied individually or concomitantly on the in vitro model (Fig. S5a, b). Therefore, further studies with all the GBCAs (gadoteridol − 0.559 kDa, gadoterate meglumine − 0.754 kDa, gadodiamide − 0.574 kDa and gadobutrol − 0.605 kDa) were performed by incubating each GBCA and NaFl in the luminal side of the EECM-BMEC-like cells and the permeability of GBCA and NaFl across monolayers of EECM-BMEC-like cells were compared. On the filters used to measure Gd permeability coefficient, we measured low permeability coefficient values with NaFl [16]. Values, given as mean ± SD, were calculated as Pe_NaFl−gadoteridol_ = 0.23 ± 0.07 × 10^− 3^cm/min, Pe_NaFl−gadodiamide_= 0.22 ± 0.04 × 10^− 3^cm/min, Pe_NaFl−gadobutrol_ = 0.25 ± 0.10 × 10^− 3^cm/min and Pe_NaFl−gadoterate meglumine_ = 0.24 ± 0.06 × 10^− 3^cm/min (Fig. 4a). Simultaneously, we measured low permeability of EECM-BMEC-like cells to all tested GBCAs, comparable to NaFl values, with mean ± SD GBCA permeability coefficients calculated from the independent experiments as Pe_gadoteridol_ = 0.32 ± 0.07 × 10^− 3^cm/min, Pe_gododiamide_= 0.34 ± 0.09 × 10^− 3^cm/min, Pe_gadobutrol_ = 0.35 ± 0.18 × 10^− 3^cm/min and Pe_gadoterate meglumine_ = 0.34 ± 0.10 × 10^− 3^cm/min (Fig. 4b).

Additionally, to confirm the integrity of the confluent monolayer, after each permeability study, the filters were immunostained for junctional proteins claudin-5, VE-cadherin or ZO-1. Uninterrupted junctional staining of all investigated molecules was detected as previously reported [16] confirming an intact monolayer of EECM-BMEC-like cells and excluding any detrimental effects of GBCAs (Fig. S5c, d). Taken together, these data show that EECM-BMEC-like cells from a cellular monolayer that prohibit free and uncontrolled passage of all GBCAs under steady state and hence prove to be a suitable model for studying whether immune cells can carry GBCA across the BBB in vitro.

### GBCA loading does not hinder immune cell migration across the BBB under physiological flow in vitro

**Fig. 5:**
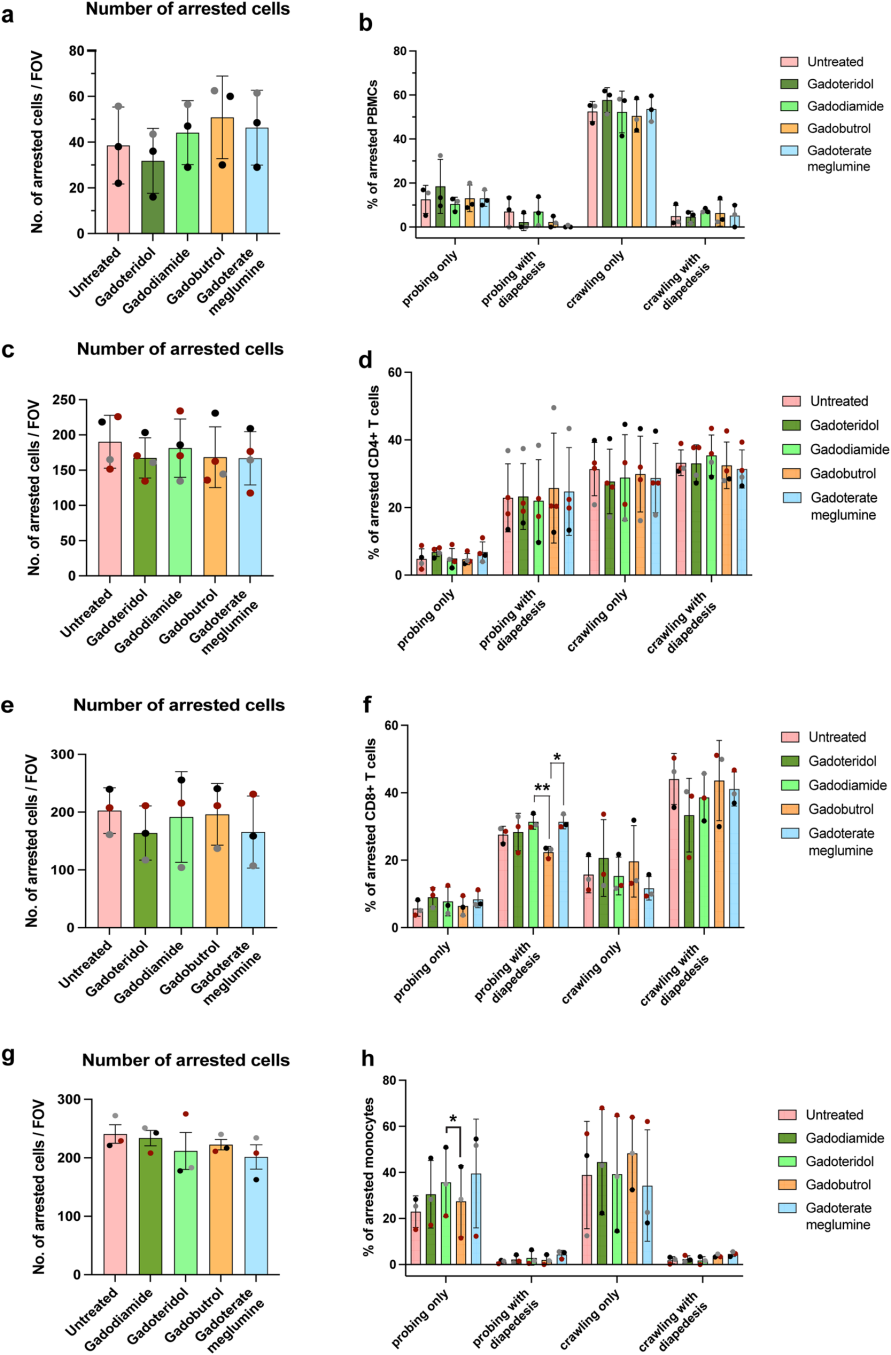
GBCA treatment does not impair the migration of immune cells across the BBB under physiological flow in vitro. PBMCs and monocytes were employed as freshly isolated cells and magnetic sorted CD4^+^ T cells and CD8^+^ T cells were additionally in vitro activated employing anti-CD3 and anti-CD28 antibodies over 5 days. These cells at 1 million cells/mL were incubated directly at a 2 mM final GBCA concentration for 1 h at 37 °C, 5% CO_2_ before experiment. Cells incubated with cell culture media only was treated as control. From atleast three independent experiments, the number of **(a)** PBMCs **(c)** in vitro activated CD4^+^ T cells, **(e)** in vitro activated CD8^+^ T cells and **(g)** monocytes arrested on TNFα/IFNγ stimulated EECM-BMEC-like cell layer and quantification of different post arrest behaviours exhibited by the arrested cells are shown in **(b)**, **(d)**,** (f)** and **(h)**. The number of arrested cells for each condition was set to 100% and the behavioural categories are shown as a fraction thereof. Each dot is the mean value of 1–3 technical replicates within an experiment, and each colour represents values from a single donor performed as an independent experiment. Data analysis was performed with one-way ANOVA with repeated measures per behavioural category followed by Tukey’s multiple comparisons test (*p* < 0.05=*, *p* < 0.01=**)

With the static experiments showing that GBCA treatment of the PBMCs and subsets did neither affect the cell surface adhesion molecule expression profile nor the ability of the immune cells to interact with recombinant human ICAM-1 or VCAM-1, we asked if these immune cells after GBCA treatment would behave as the untreated cells under flow conditions. Immune cells are exposed to shear forces in the blood stream and these forces also strengthen the interaction between the immune cell integrin - endothelial ligand binding [35]. Therefore, we investigated the interactions between circulating immune cells and cytokine stimulated EECM-BMEC-like cells mimicking an inflamed BBB, under physiological flow. Initially, we studied the migration behaviour of PBMCs with or without GBCA treatment across BBB endothelial cells. An additional movie file shows PBMCs interacting with the in vitro BBB model (see Additional file 2). We observed that GBCA treatment did not affect the arrest, post-arrest dynamic behaviour and diapedesis of PBMCs across the BBB under physiological flow (terminologies defined in Materials and Methods; Fig. 5a, b, Fig. S6a).

Next, we investigated the migratory behaviour of individual immune cell subsets upon GBCA treatment as the frequency of each subpopulation of immune cells is different in PBMCs which may lead to the behaviour of immune cells present at lower frequencies being masked by behaviours exhibited by immune cells present in higher numbers. We employed in vitro activated CD4^+^ and CD8^+^ T cells as these are known to cross the BBB in vivo. In case of monocytes, cells freshly isolated from PBMCs where used. Comparing the number of CD4^+^ T cells, CD8^+^ T cells and monocytes arrested on the EECM-BMECs under physiological flow, no significant difference was observed between untreated immune cells and immune cells that were incubated with the different contrast agents (Fig. 5c, e, g). Percentage of arrested cells that either probed or crawled followed by diapedesis or not is shown in Fig. 5d, f and h. Quantification of post-arrest behaviour of all arrested immune cells was quantified and categorised as followed: crawling (all crawling), probing (all probing), performing diapedesis (all diapedesis), stationary or detached which is shown in Fig. S6. In case of CD8^+^ T cells, incubation with gadodiamide and gadoterate meglumine led to a significantly higher total probing behaviour (All Pobing) compared to untreated CD8^+^ T cells (Fig. S6c). However, this did not result in significantly increased diapedesis. In case of monocytes, there was a significant increase in the percentage of cells migrating across the EECM-BMEC-like cell layer (All Diapedesis) after gadobutrol-loading compared to untreated cells (Fig. S6d). Additional movie files show the behaviour of CD4^+^ T cells, CD8^+^ T cells and monocytes on the in vitro BBB model (Additional files 3–5). In case of NK cells and B cells, no quantifiable cell arrest was observed on to the EECM-BMEC-like cell layer. Even though, in vitro IL-2 activated NK cells were arrested on to the cell layer, it was observed that the bound cells induced endothelial cell death in the areas around the site of NK cell arrest. Therefore, no further analysis relating to their post arrest behaviour could be performed.

After comparing the number of arrested immune cells and the different post arrest behaviours exhibited by the immune cell populations incubated with various GBCAs with the EECM-BMEC-like cells, we did not observe reduced post-arrest dynamic interactions or reduced diapedesis of the immune cells suggesting that contrast agents could enter the CNS as cargo of immune cells crossing the BBB.

## Discussion

In this study, we propose a novel mechanism of Gd deposition in the brain via the BBB and report, for the first time measurable amounts of Gd in various blood cell populations. We have further shown that GBCA loading did not affect PSGL-1, α4β1-integrin and αLβ2-integrin cell surface expression on the immune cells nor hindered the interaction of the immune cells with recombinant VCAM-1 or ICAM-1. Moreover, we employed a well-established in vitro BBB model to successfully demonstrate that GBCA loaded circulating immune cells can extravasate across the BBB under physiological flow in vitro in similar rates to untreated immune cells.

The BBB has low vesicular activity and prohibits free paracellular diffusion of water-soluble molecules by its complex and continuous tight junction. Hydrophilic molecules such as sucrose (340 Da, radius 0.5 nm) or inulin (5000 Da, radius 1.5 nm) have been used to investigate BBB barrier properties of BBB models [36, 37]. GBCAs are hydrophilic in nature and are marketed as either ionic or non-ionic agents. The ionic agents have an overall negative charge while the non-ionic agents are neutral [38]. Additionally, the high molecular weight of GBCAs (> 500 Da) also restricts GBCAs from passively traversing the BBB under steady state conditions [39]. It is thus generally accepted that GBCAs do not cross an intact BBB [32] and consequently Gd-enhancing lesions are useful markers to assess diagnosis and prognosis of MS pathology [34]. However, Gd depositions were observed associated with brain capillaries in patients without intracranial abnormalities that challenged the BBB impermeability to GBCA [7] and later, a more detailed study in rats showed the Gd localisation to basal lamina and perivascular Virchow-Robin space of the brain microvessels [40]. However, the exact mechanism of GBCA access to the brain remains unknown.

Building on our earlier findings of detecting Gd in the WBCs of patients administered gadoterate meglumine during MRI [15], we were interested in exploring if circulating immune cells after intravenous GBCA administration might take up GBCAs and carry them as cargo across the BBB and into the brain. Additionally, a previous study demonstrated GBCA uptake into WBCs after incubation for up to 3 h at a 5 mM concentration [30]. Initially, we aimed to determine if the different immune subpopulations present in the blood could be loaded with GBCAs. Employing the SC-ICP-MS technique, we measured Gd in the peripheral blood cell populations after in vitro treatment with both linear and macrocyclic GBCAs. Given the varying proportions of blood cell populations [41], it is noteworthy that certain populations exhibited a higher Gd loading compared to others. Monocytes comprising approximately 7% of total blood cells, showed the highest Gd measurements, followed by NK cells that constitute around 3.5% of blood cells. Circulating CD4^+^ and CD8^+^ T cells and B cells, forming significant proportions of the blood immune cells, also showed Gd loading. Moreover, in vitro activated CD4^+^ and CD8^+^ T cells showed similar Gd loading as circulating T cells. We also confirmed Gd loading in neutrophils which constitute the largest proportion of granulocytes in blood. These results are of great interest as peripherally activated T cells and monocytes are extensively studied for migration into brain tissues in certain conditions that involve BBB dysfunction, including in MS, and patients with these conditions undergo multiple contrast enhanced MRIs as part of disease monitoring.

Furthermore, from the panel of GBCAs we tested, we observed that gadodiamide treatment led to a higher Gd loading in most of the cell populations. As gadodiamide was the only linear GBCA we tested, and the transport mechanism of the contrast agents into the cells is unknown, it is unclear whether the observed result is due to structural differences between the GBCAs. Due to the majority of GBCA related NSF cases attributed to use of gadodiamide, its usage has been restricted by the European Medicines Agency (EMA). Even though gadodiamide is still approved by U.S. Food and Drug Administration (FDA), macrocyclic agents are preferably employed in the clinic.

Other studies have investigated the effects of treatment of immune cells with both linear and macrocyclic GBCAs (reviewed in [42]). Friebe et al. 2018 [43] showed that PBMCs treated with linear and macrocyclic GBCAs at 2 mM concentration for one hour did not lead to genotoxic effects in the cells, determined by induction of γH2AX foci by both linear and macrocyclic agents. Additionally, the study also reported that 24 h incubation of the PBMCs with 2 mM gadodiamide lead to significant increase in apoptosis of the cells, but not with the macrocyclic agents studied.

To understand how GBCA treatment may affect immune cell extravasation across the BBB, we focussed on the factors essential for the process. A key factor is the cell surface expression of the adhesion molecule PSGL-1, along with integrins α4β1 and αLβ2 on the immune cells that interact with the different endothelial adhesion molecules P-selectin, VCAM-1 and ICAM-1 at the BBB in a sequential manner [14]. Our results show that the GBCA presence in the immune cells did not alter the cell surface expression profile of key adhesion molecules that supports immune cell extravasation across the BBB. Using flow cytometry, with markers for the different integrin subunits α4, β1, αL, β2 and for PSGL-1 additional to the cell specific markers, we were able to study the cell surface expression profile of the different molecules on different immune cell subsets directly from the PBMCs as isolated from the healthy donor buffy coats. This allowed us to maintain the proportions of different cell populations similar to as present in peripheral blood. Since some cell populations showed high and low integrin expression, we additionally gated for cells expressing high or low levels of integrins to investigate if exposure to the contrast agents might alter the expression levels of specific integrins on the cells within these populations. In all the cases, we confirmed that GBCA exposure had no significant effect on the expression of the investigated adhesion molecules.

An ‘active’ conformational state of immune cell integrins is crucial for interaction with their endothelial ligands, enabling successful arrest to the BMECs and possible extravasation of immune cells [31]. In our study, we observed that both untreated and GBCA loaded immune cells bound in comparable numbers to recombinant ICAM-1 and VCAM-1. This indicates that GBCA presence did not affect the ability of the immune cell subsets to engage with the key molecules in the extravasation process across the BBB under static conditions.

As shear forces are present in the bloodstream with significant impact on immune cell interactions with the BBB [35, 44], the dynamic interaction of immune cells with the BBB was investigated under physiological flow. Here, the flow rates of the medium containing the immune cells were set to generate the physiological shear present in the post capillary venules [45]. In our experiments, we confirmed that EECM-BMEC-like cells form a diffusion barrier for the GBCAs tested. Employing the EECM-BMEC-like cell in vitro BBB model, we confirmed that GBCA-loaded immune cells extravasated across EECM-BMEC-like cell monolayers at similar rates to untreated immune cells under physiological flow conditions. Collectively, our findings demonstrate that GBCA loading did not alter dynamic immune cell behaviour at the BBB, and the immune cell migration across the BBB is comparable to untreated immune cells in vitro under physiological conditions. Since the migrating immune cells could be loaded with GBCA, this migration could facilitate transport of GBCA from peripheral blood to the brain potentially leading to its deposition in brain border zones or the parenchyma. Concurrently, studies have reported deposition of Gd in the capillary basement membranes and perivascular spaces, with electron microscopy techniques validating its presence, both in animal studies [40] and post mortem human tissue analysis [46, 47], even in the absence of intracranial abnormalities and presumably an intact BBB [7]. With our results indicating immune cells taking GBCA as cargo across BBB into the perivascular space, it is tempting to speculate that this could be responsible for the Gd deposits observed in the aforementioned studies in association with the BBB. Therefore, in case of both T cells and monocytes, the GBCA possibly brought in as cargo can access the perivascular spaces and further fates of the molecule is to be studied. Activated T cells can cross the intact BBB even in the absence of neuroinflammation in the context of CNS immune surveillance, however they remain restricted to CNS border zones such as the perivascular spaces or the CSF-filled subarachnoid space [48–50]. This may lead to GBCA release into the CSF. In contrast, monocytes do not cross the non-inflamed BBB, but can cross an inflamed BBB [10]. Other immune cell subsets have also been shown to extravasate across the BBB depending on the conditions – for example, neutrophils in meningitis and ischemic stroke, B cells in MS (summarised in [10, 51]) and NK cells in MS [52] and ischemic stroke [53].

In conclusion, our study provides convincing evidence that circulating immune cells can load GBCA and that GBCA load does not affect their migration across the BBB under physiological flow in vitro. Especially, activated T cells can cross the BBB also in the context of CNS immune surveillance but then remain restricted to CNS border zones. These findings could explain reports of Gd deposition in perivascular spaces of brain associated vessels. However, in this study we have not specifically assessed if GBCA may affect other cell functions like cytokine expression or long-term viability. The prolonged retention of GBCA in immune cells is also to be assessed, as we speculate, based on our unpublished preliminary observations, that GBCA will be gradually cleared from the cells over time. If the clearance continues to occur after the immune cells have migrated across the BBB, it might also lead to release of GBCA around blood vessels or in tissues as previously reported [54]. In the present study, we focused on evaluating the ability of human immune cells to transport Gd across the BBB using an established in vitro human BBB model. In vivo studies employing immunocompetent animal models will be essential to further elucidate this proposed novel route of Gd entry into the brain under physiological conditions. With regard to the GBCA deposited in the tissues, it is speculated that over longer periods of time, Gd^3+^ could dissociate from the chelate depending on the local microenvironment [55] and interact with macromolecules present [56]. Furthermore, as we have previously observed a subset of the patients showing a higher GBCA loading in WBCs [15], it needs is to be investigated if those patients are at a higher risk to develop Gd depositions within the body and specifically in the brain.

## Electronic supplementary material

Below is the link to the electronic supplementary material.

Additional file 1: Additional figures (1-6) and Tables (1-4) supporting the manuscript given as a word file (.doc).

Additional file 2: **Timelapse video of in vitro live cell imaging of PBMCs interacting with EECM-BMEC-like cells under physiological flow**. EECM-BMEC-like cells were cultured at a density of 75,000 cells/cm^2^ in ibidi µ-Dishes coated with collagen IV (400 mg/mL) and fibronectin (100 mg/mL) in water. After 24 h of adding cells, EECM-BMEC-like cells were stimulated with 0.1 ng TNFa + 2IU/mL IFNg diluted in conditioned media from SMLC of the same donor for 16 h at 37 °C, 5% CO_2_. A flow chamber was added on to the ibidi µ-Dish over the EECM-BMEC-like cells and flow applied. PBMCs at 1 million cells/mL were superfused over the EECM-BMEC-like cells and allowed to accumulate in the first four minutes under 0.1 dynes/cm^2^ shear stress (accumulation phase). This was followed by increasing the shear stress to 1.5 dynes/cm^2^ until the end of the experiment (shear phase). The red arrow on the top right shows the direction of flow during the experiment and the elapsing time (mm: ss format) is shown on top left. During the recording, images were captured every 10 s. In the video, the green circle shows a probing cell, the yellow circle shows a crawling cell, and the magenta circle shows a cell performing diapedesis

Additional file 3: **Timelapse video of in vitro live cell imaging of CD4**^**+**^**T cells interacting with EECM-BMEC-like cells under physiological flow**. EECM-BMEC-like cells were cultured at a density of 75,000 cells/cm^2^ in ibidi µ-Dishes coated with collagen IV (400 m g/mL) and fibronectin (100 m g/mL) in water. After 24 h of adding cells, EECM-BMEC-like cells were stimulated with 0.1 ng TNFa + 2IU/mL IFNg diluted in conditioned media from SMLC of the same donor for 16 h at 37 °C, 5% CO_2_. A flow chamber was added on to the ibidi µ-Dish over the EECM-BMEC-like cells and flow applied. CD4^+^ T cells at 1 million cells/mL were superfused over the EECM-BMEC-like cells and allowed to accumulate in the first four minutes under 0.1 dynes/cm^2^ shear stress (accumulation phase). This was followed by increasing the shear stress to 1.5 dynes/cm^2^ until the end of the experiment (shear phase). The red arrow on the top right shows the direction of flow during the experiment and the elapsing time (mm: ss format) is shown on top left. During the recording, images were captured every 10 s. In the video, the green circle shows a probing cell, the yellow circle shows a crawling cell, and the magenta circle shows a cell performing diapedesis

Additional file 4: **Timelapse video of in vitro live cell imaging of CD8**^**+**^**T cells interacting with EECM-BMEC-like cells under physiological flow**. EECM-BMEC-like cells were cultured at a density of 75,000 cells/cm^2^ in ibidi µ-Dishes coated with collagen IV (400 mg/mL) and fibronectin (100 mg/mL) in water. After 24 h of adding cells, EECM-BMEC-like cells were stimulated with 0.1 ng TNFa + 2IU/mL IFNg diluted in conditioned media from SMLC of the same donor for 16 h at 37 °C, 5% CO_2_. A flow chamber was added on to the ibidi µ-Dish over the EECM-BMEC-like cells and flow applied. CD8^+^ T cells at 1 million cells/mL were superfused over the EECM-BMEC-like cells and allowed to accumulate in the first four minutes under 0.1 dynes/cm^2^ shear stress (accumulation phase). This was followed by increasing the shear stress to 1.5 dynes/cm^2^ until the end of the experiment (shear phase). The red arrow on the top right shows the direction of flow during the experiment and the elapsing time (mm: ss format) is shown on top left. During the recording, images were captured every 10 s. In the video, the green circle shows a probing cell, the yellow circle shows a crawling cell, and the magenta circle shows a cell performing diapedesis

Additional file 5: **Timelapse video of in vitro live cell imaging of monocytes interacting with EECM-BMEC-like cells under physiological flow**. EECM-BMEC-like cells were cultured at a density of 75,000 cells/cm^2^ in ibidi µ-Dishes coated with collagen IV (400 mg/mL) and fibronectin (100 mg/mL) in water. After 24 h of adding cells, EECM-BMEC-like cells were stimulated with 0.1 ng TNFa + 2IU/mL IFNg diluted in conditioned media from SMLC of the same donor for 16 h at 37 °C, 5% CO_2_. A flow chamber was added on to the ibidi µ-Dish over the EECM-BMEC-like cells and flow applied. Monocytes at 1 million cells/mL were superfused over the EECM-BMEC-like cells and allowed to accumulate in the first four minutes under 0.1 dynes/cm^2^ shear stress (accumulation phase). This was followed by increasing the shear stress to 1.5 dynes/cm^2^ until the end of the experiment (shear phase). The red arrow on the top right shows the direction of flow during the experiment and the elapsing time (mm: ss format) is shown on top left. During the recording, images were captured every 10 s. In the video, the green circle shows a probing cell, the yellow circle shows a crawling cell, and the magenta circles show cells performing diapedesis

## Data Availability

No datasets were generated or analysed during the current study.

## Abbreviations

Gd: Gadolinium
GBCA: Gadolinium-based contrast agent
BBB: Blood-brain barrier
SC-ICP-MS: Single-cell inductively coupled mass spectrometry
MRI: Magnetic resonance imaging
NSF: Nephrogenic systemic fibrosis
CNS: Central nervous system
PSGL-1: P-selectin glycoprotein-1
VCAM-1: Vascular cell adhesion molecule-1
ICAM-1: Intercellular adhesion molecule − 1
BMEC-like cells: Brain microvascular endothelial cell-like cells
hiPSCs: Human induced pluripotent stem cells
EECM: Extended endothelial cell culture method
TEER: Transendothelial electrical resistance
ZO-1: Zona occludens-1
PBMC: Peripheral blood mononuclear cell
FBS: Fetal bovine serum
WBC: White blood cell
SMLC: Smooth muscle-like cell
CM: Conditioned media
