# Supplementary material for: Identifying a potential role of immune cells in gadolinium deposition within the brain
Source: Fluids Barriers CNS. 2025 Jul 29;22:80. doi: 10.1186/s12987-025-00674-5 (PMC12306026; doi:10.1186/s12987-025-00674-5)
Supplement: Supplementary file 1 — Additional file 1: Additional figures (1-6) and Tables (1-4) supporting the manuscript given as a word file (.doc). [file 12987_2025_674_MOESM1_ESM.docx]

**Identifying a potential role of immune cells in gadolinium deposition within the brain**

Dixy Parakkattel^a,b^, Nico Ruprecht^a,b^, Peter Broekmann^c^, Sarah Guimbal^d^, Chiara Stüdle^d^, Sasha Soldati^d^, Johannes T. Heverhagen^a,b^, Britta Engelhardt^d,*^, Hendrik von Tengg-Kobligk^a,b,*^

^a^Department of Diagnostic, Interventional and Pediatric Radiology, Inselspital, University of Bern, Switzerland, ^b^Department of BioMedical Research, University of Bern, Bern, Switzerland, ^c^Department of Chemistry, Biochemistry and Pharmaceutical Sciences, University of Bern, Bern, Switzerland, ^d^Theodor Kocher Institute, University of Bern, Bern, Switzerland

**^5^Correspondence:**

Britta Engelhardt

Theodor Kocher Institute, University of Bern, Bern, Switzerland

E-Mail: britta.engelhardt@unibe.ch

Hendrik von Tengg-Kobligk

Diagnostic, Interventional and Pediatric Radiology, Inselspital, University of Bern, Bern, Switzerland

E-mail: Hendrik.vonTengg@insel.ch

**Figure S1**

**Fig. S1: GBCA loading of immune cells**. Gd concentration measured in **(a)** CD4^+^ T cells and **(b)** CD8^+^ T cells polyclonally activated by anti-CD3 and anti-CD28 treatment *in vitro* over a 5-day period as described in the Materials and Methods are shown. Additionally, Gd concentration measured in **(c)** freshly isolated neutrophils are shown. Isolated neutrophils and *in vitro* activated T cells at 1 million cells/mL cells were incubated at a 2 mM final concentration of GBCA for 1 hour at 37°C, 5% CO_2_. Gd concentration in cells was measured using SC-ICP-MS and is shown in attograms/cell (ag/cell). Each dot is the mean value of 2-3 technical replicates, and each colour represent values from a single donor performed as an independent experiment. The donor cells used for the three (or two in case of neutrophils) independent experiment varies between the graphs. Analysis performed using One-way ANOVA with repeated measures followed by Tukey’s multiple comparisons test and shown as mean ± SD (p<0.05=*).

**Figure S2**

**
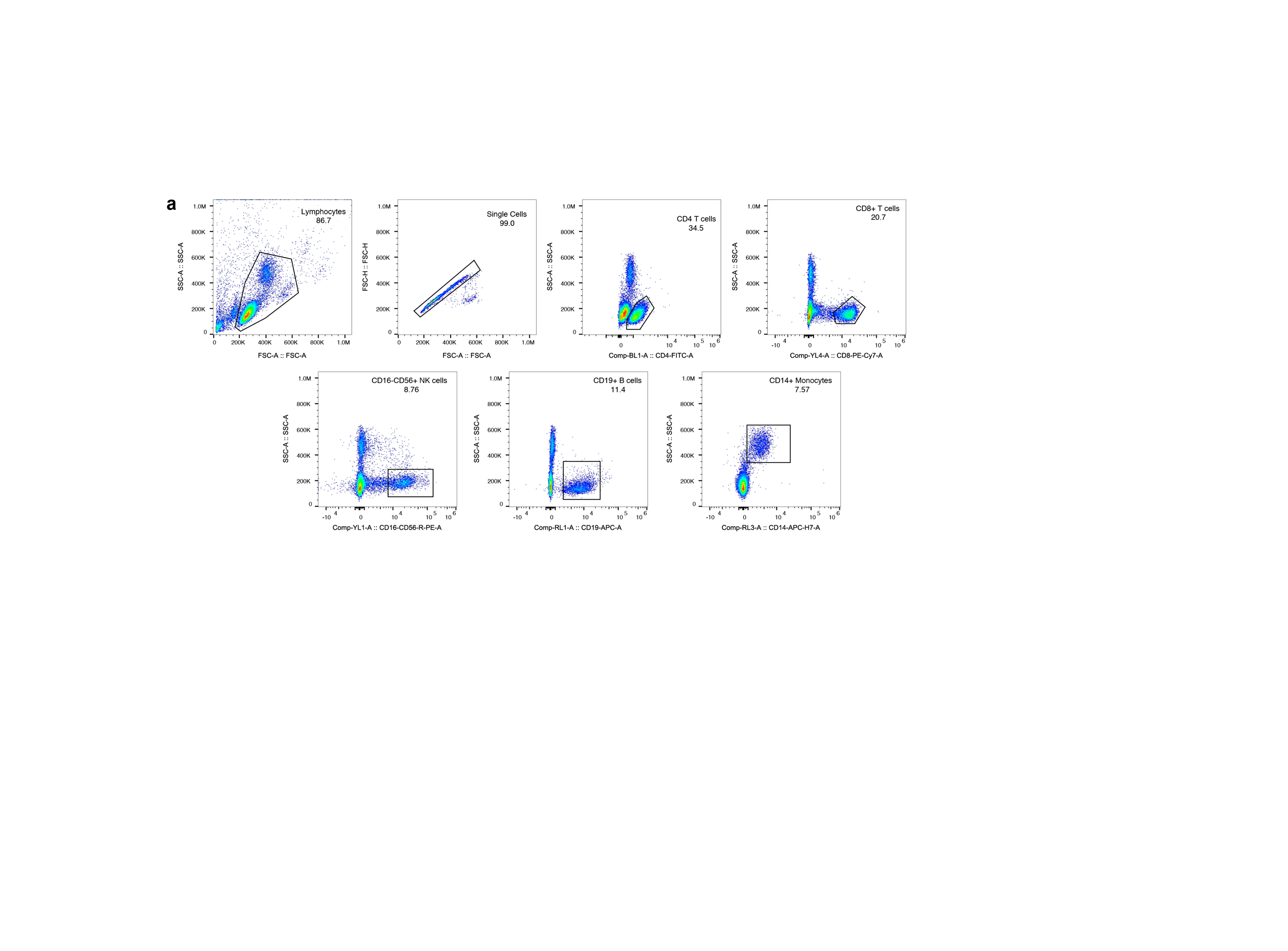
**

**
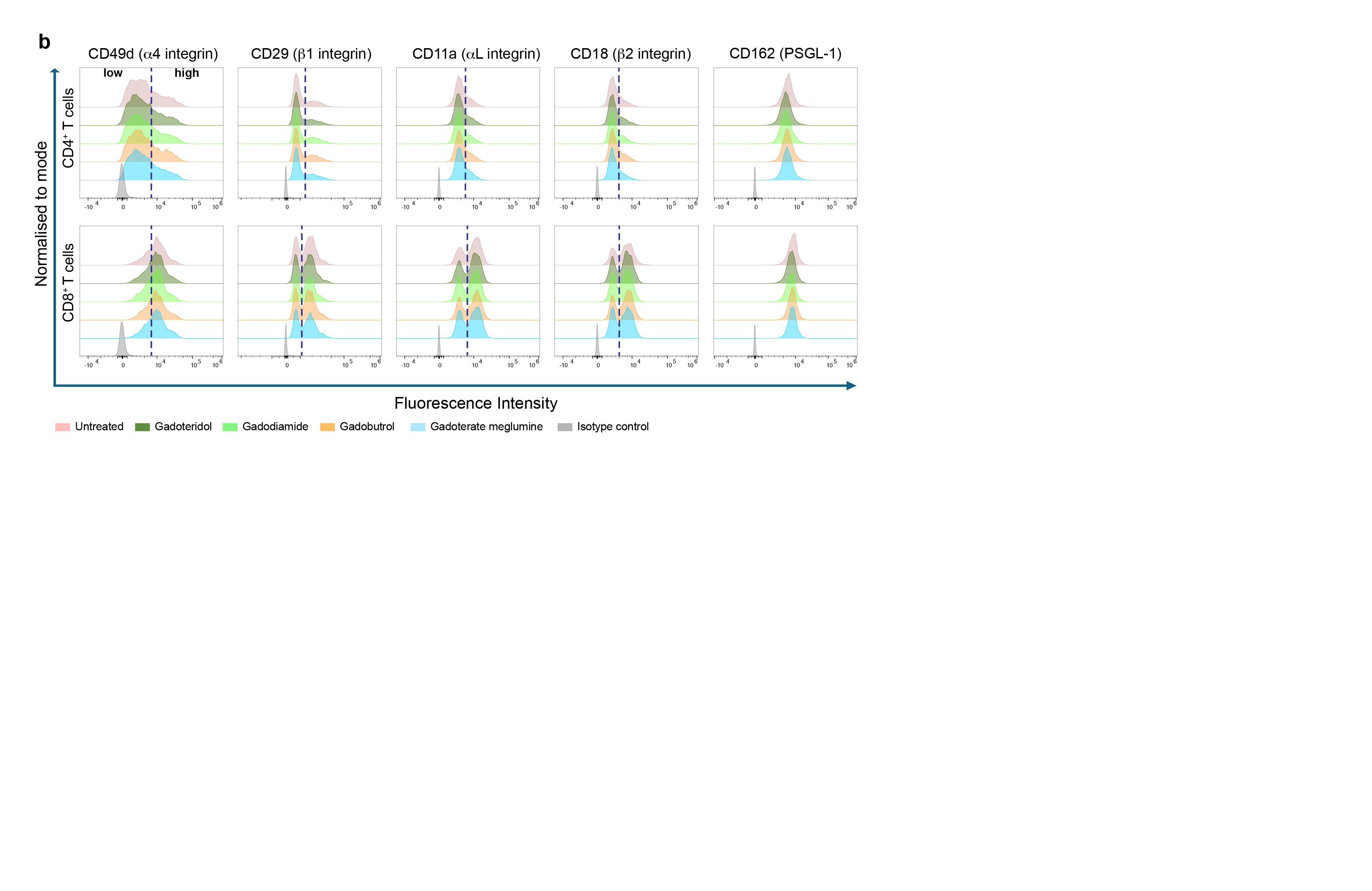
**

**Fig. S2: GBCA treatment does not affect cell surface adhesion molecule expression. (a)** Representative gating strategy for the multicolour flow cytometry analysis of PSGL-1, α4 -, β1- , αL- and β2- integrin cell-surface expression on CD4^+^ T cells, CD8^+^ T cells, monocytes, NK cells and B cells. PBMCs at 1 million cells/mL cells were incubated at a 2 mM final GBCA concentration for 1 hour at 37°C, 5% CO_2_. Cells incubated with cell culture media only was treated as control. **(b)** Gating strategy used to quantify percentage of cells with high or low integrin expression and to calculate ΔMFI of each peak in CD4^+^ T cells and CD8^+^ T cells. Isotype control is shown in grey. Percentage of cells with high or low expression of integrins in **(c)** CD4^+^ T cells and **(d)** CD8^+^ T cells from one donor cells as representative of four independent experiments shown. ΔMFI of peaks from **(e)** CD4^+^ T cells, **(f)** CD8^+^ T cells, **(g)** monocytes, **(h)** NK cells and **(i)** B cells from four independent experiments using four different donor cells with each dot representing data from single donor. Analysis performed using One-way ANOVA with repeated measures followed by Tukey’s multiple comparisons test and shown as mean ± SD.

**Figure S3**

**
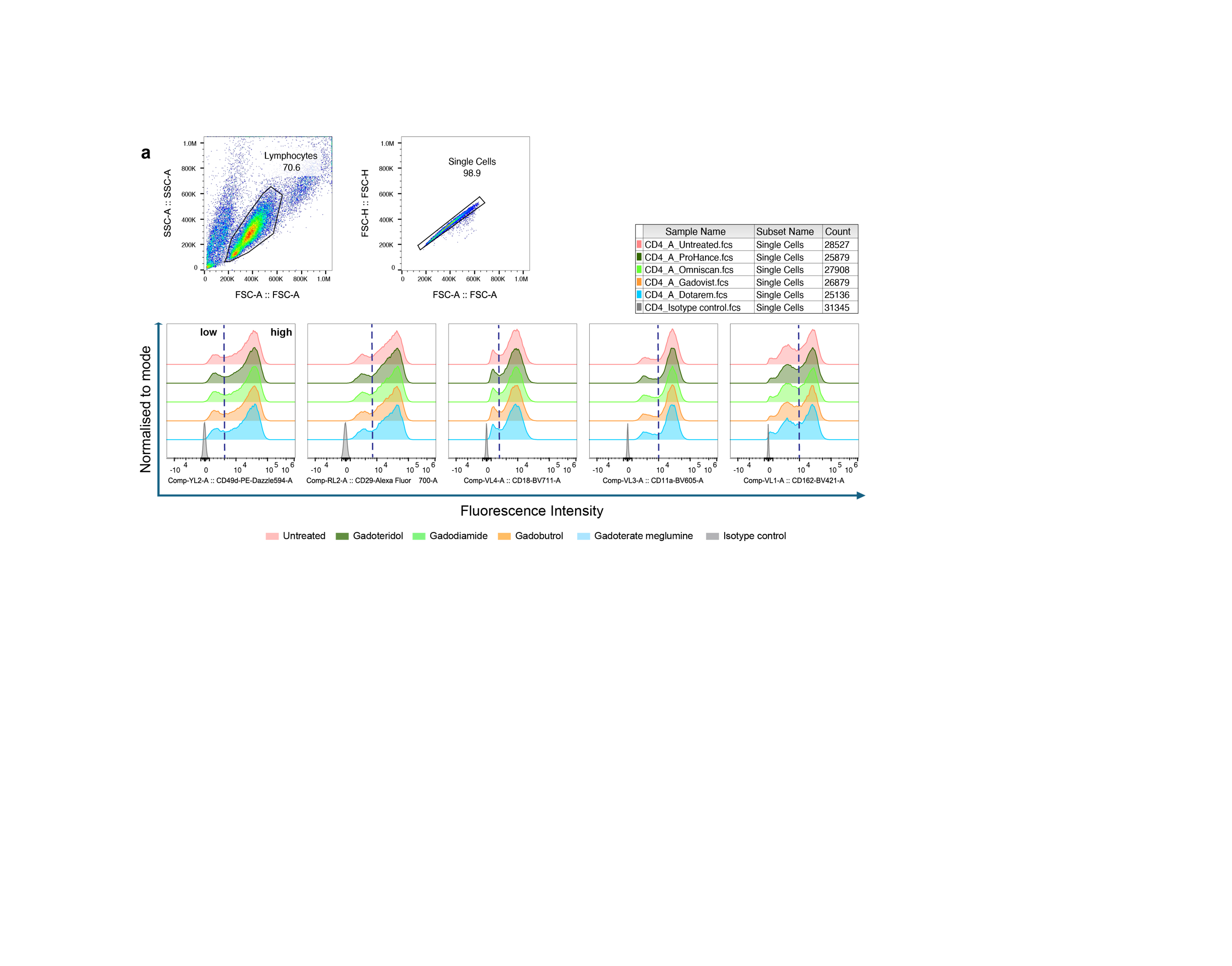
**

**
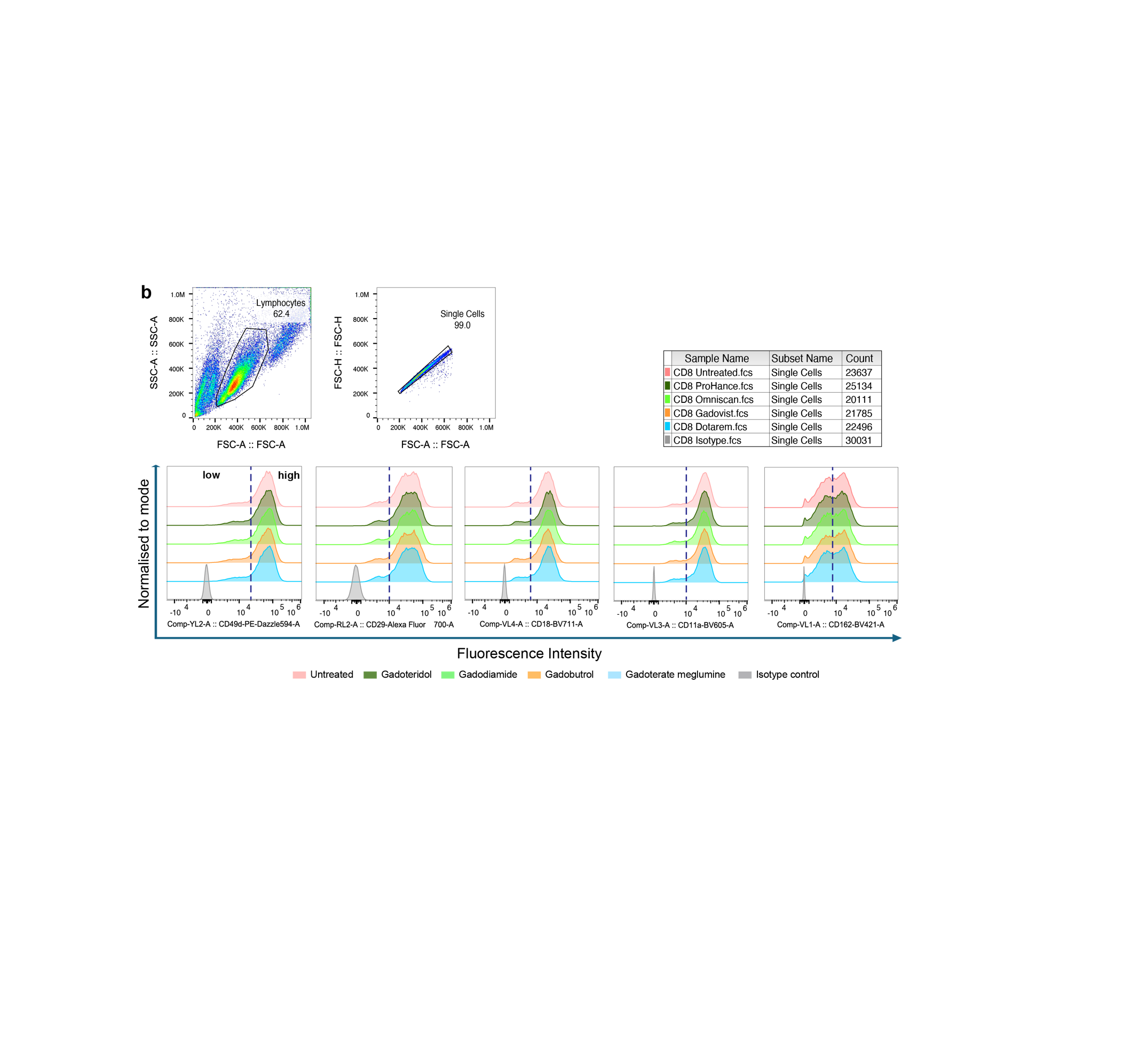
**

**Fig. S3: GBCA treatment does not alter cell surface adhesion molecule expression on *in vitro* activated CD4^+^ and CD8^+^ T cells.** Representative gating strategy and expression profile from multicolour flow cytometry analysis of PSGL-1, α4 -, β1- , αL- and β2- integrin cell-surface expression on *in vitro* activated **(a)** CD4^+^ and **(b)** CD8^+^ T cells with agonistic antibodies against CD3 and CD28 over a 5-day period. Isotype control is shown in grey. Activated T cells at 1 million cells/mL cells were incubated at a 2 mM final GBCA concentration for 1 hour at 37°C, 5% CO_2_. Cells incubated with cell culture media only was treated as control. Gating strategy used to quantify percentage of cells with high or low integrin expression and to calculate ΔMFI of each peak in CD4^+^ T cells and CD8^+^ T cells. Percentage **(c)**, **(d)** and ΔMFI **(e)**, **(f)** of PSGL-1, α4 -, β1- , αL- and β2- integrins high and low expressing CD4^+^ T cells and CD8^+^ T cells from one donor cells representative of three independent experiments given.

**Figure S4**

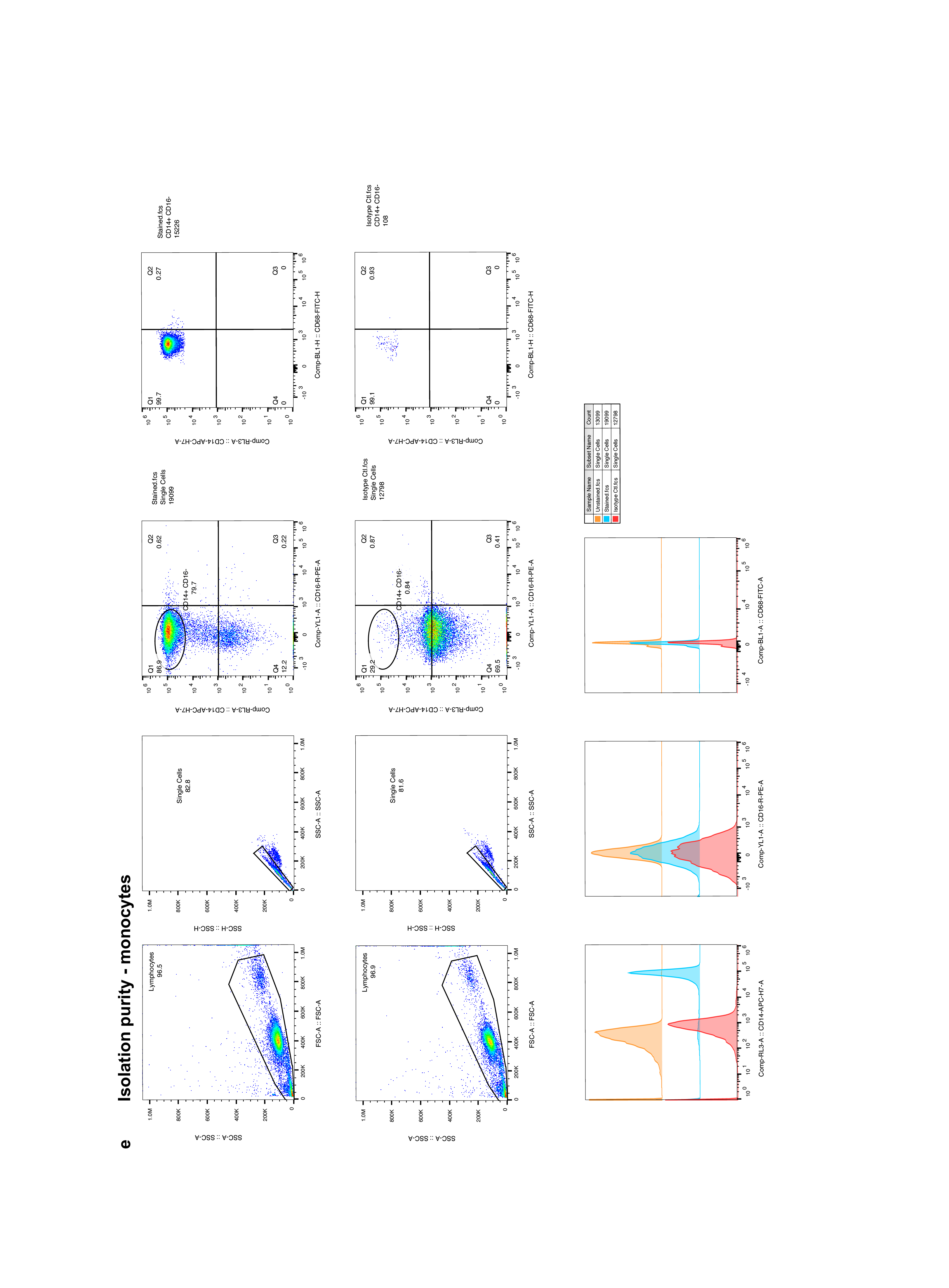

**Fig. S4**: **Cell purity analysis**. **(a)** CD4^+^ T cells, **(b**) CD8^+^ T cells, **(c)** B cells, **(d)** NK cells, and **(e)** monocytes were magnetic isolated from PBMCs while **(f)** neutrophils were magnetic isolated from the granulocyte fraction during PBMC isolation from buffy coat using Ficoll-Paque Plus density gradient. All isolations were performed using the specific EasySep isolation kits from StemCell Technologies as described in Materials and Methods and the purity check as shown was performed once for each immune cell subset.

**Figure S5**

**
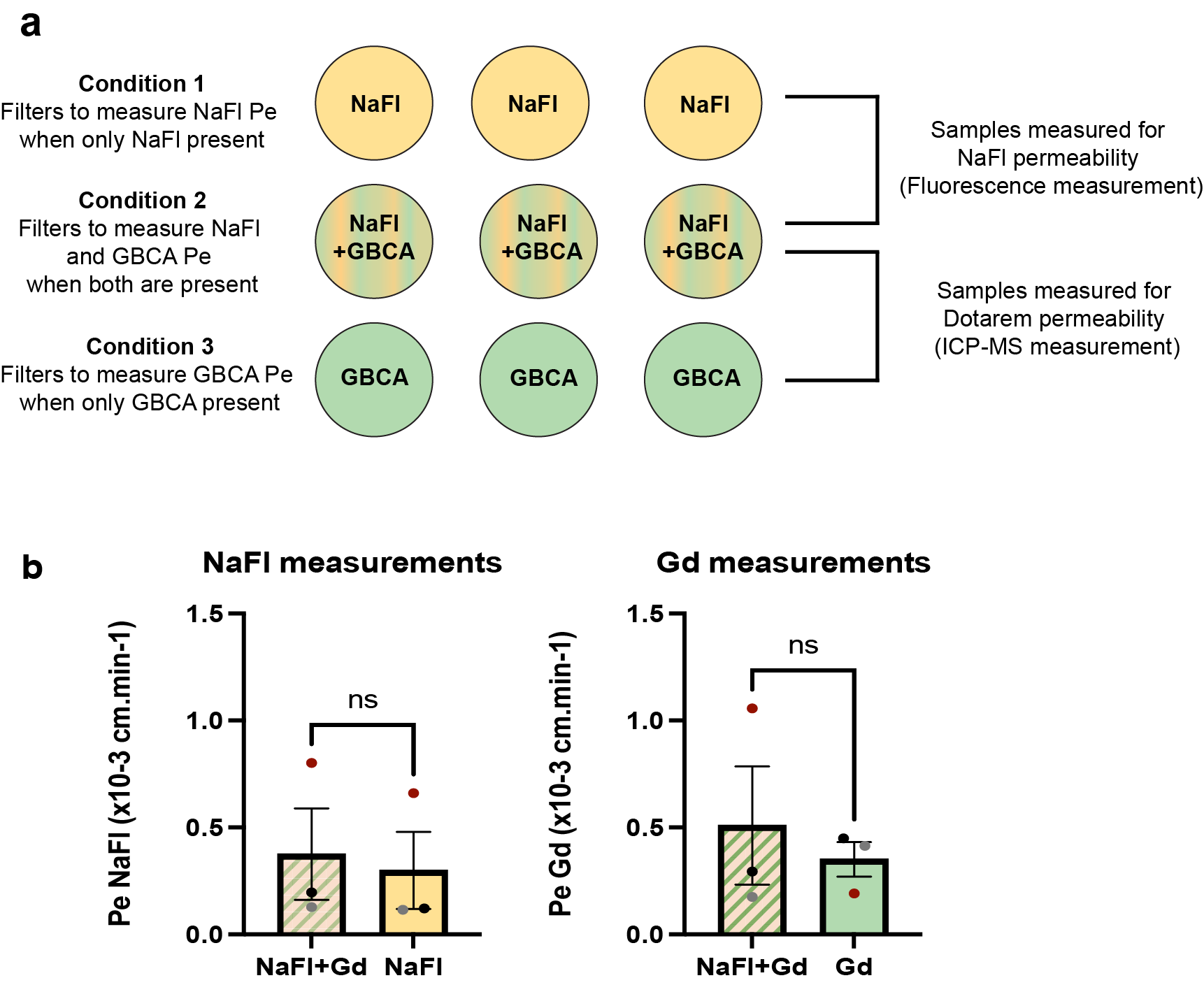
**

**
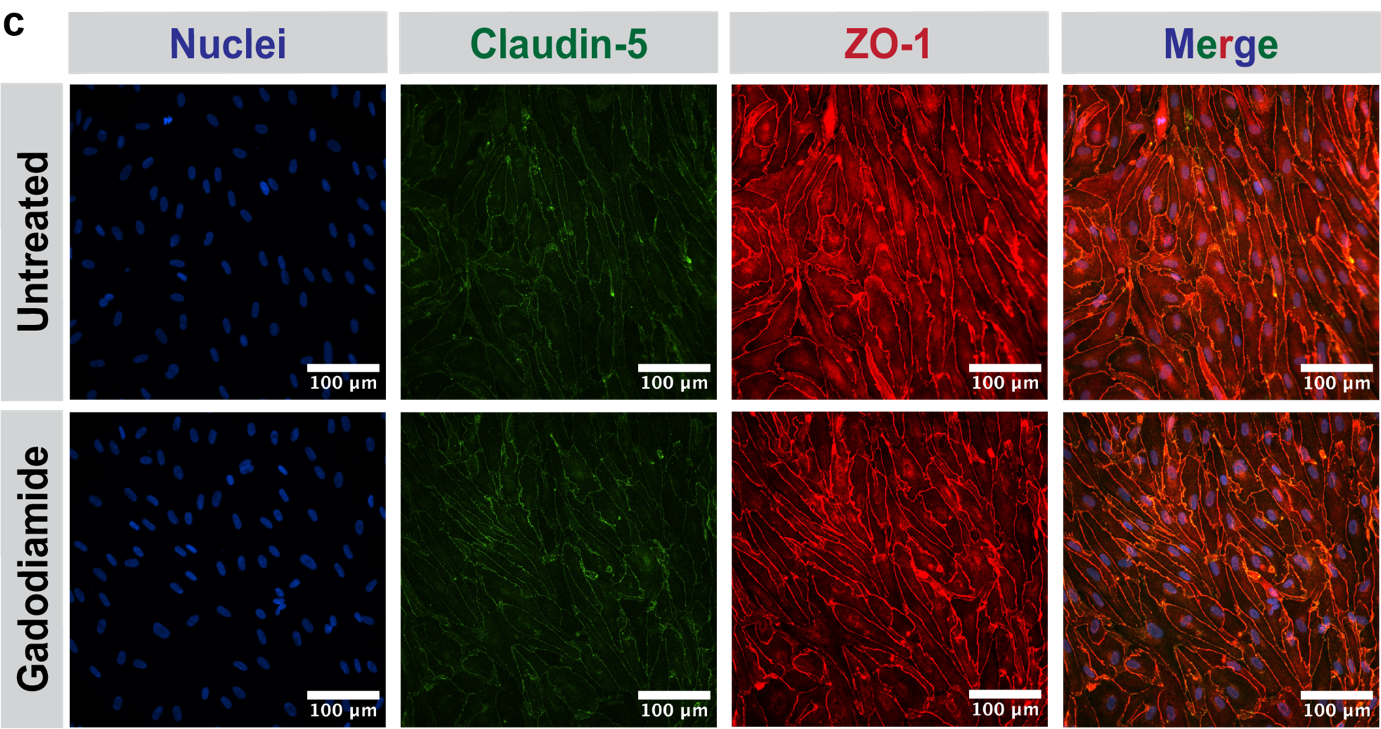
**

**
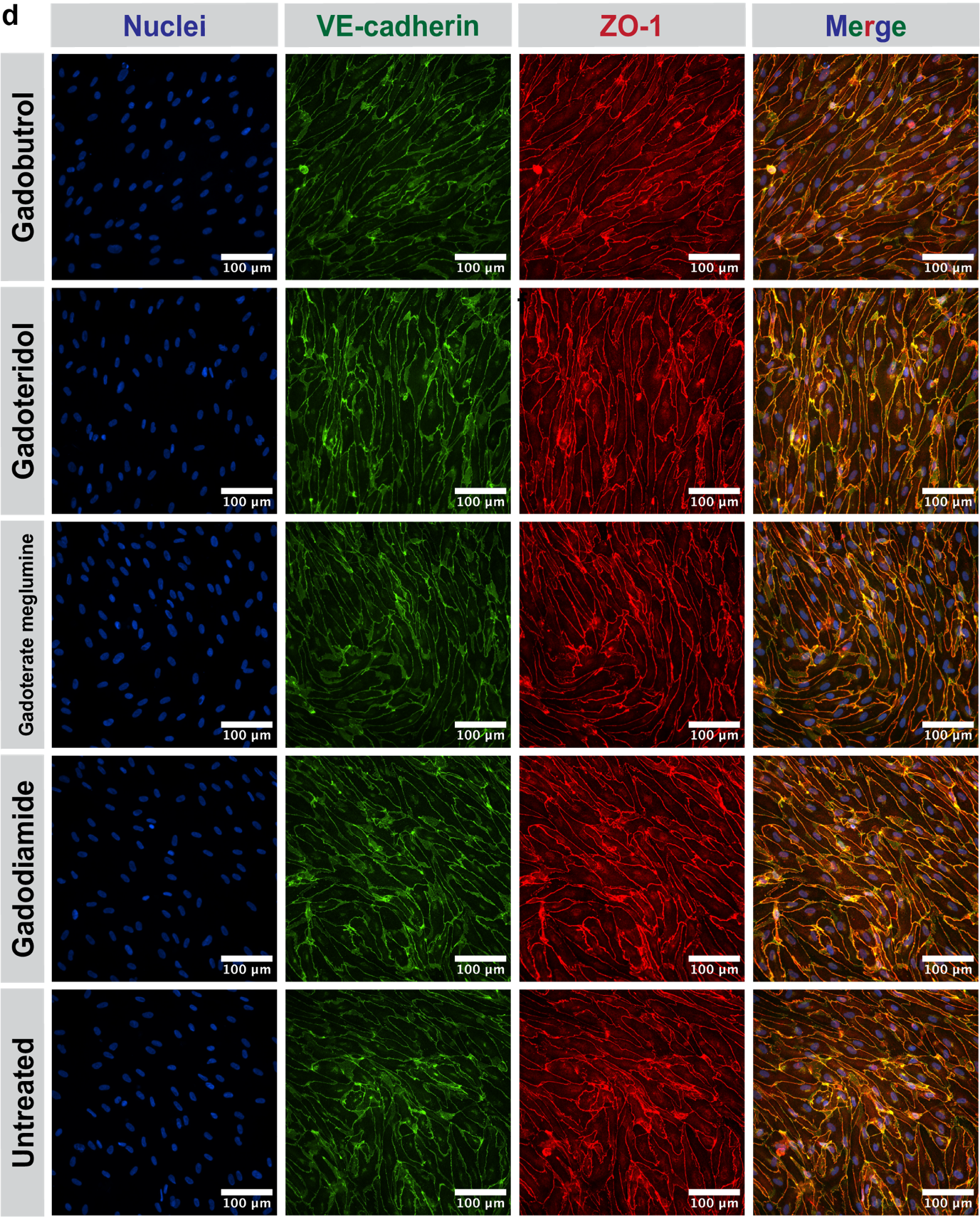
**

**Fig. S5**: **Characterization of barrier properties of EECM-BMEC-like cells for NaFl and GBCA.** **(a)** The permeability for NaFl (376 Da) and Dotarem (754 Da) were measured in three different conditions in one experiment with three filters per condition to investigate if simultaneous application of GBCA and NaFl affected the EECM-BMEC-like cell monolayer (Condition A with only NaFl (n=3), Condition B with NaFl and Dotarem (n=3) and Condition 3 with Dotarem only n=3) **(b)** Permeability coefficients measured for NaFl and Dotarem. Paired t-test was employed to check for significant difference in the calculated permeability coefficients. **(c)** EECM-BMEC-like cells grown on Transwell filters for 6 days and on day 6, filters were either treated with Omniscan at 2 mM for 1 hour at 37°C, 5% CO_2_ or with media only (Untreated) and staining for DAPI, Claudin-5 and ZO-1 performed as described in Materials and Methods. Scalebar = 100 µm. **(d)** EECM-BMEC-like cells grown on Transwell filters for 6 days and on day 6, filters were either treated with Gadovist, ProHance, Dotarem, Omniscan at 2 mM for 1 h at 37°C, 5% CO_2_ or with media only (Untreated) and staining for DAPI, VE-cadherin and ZO-1 performed as described in Materials and Methods. Scalebar = 100 µm. The staining as shown was performed once.

**Figure S6**

**
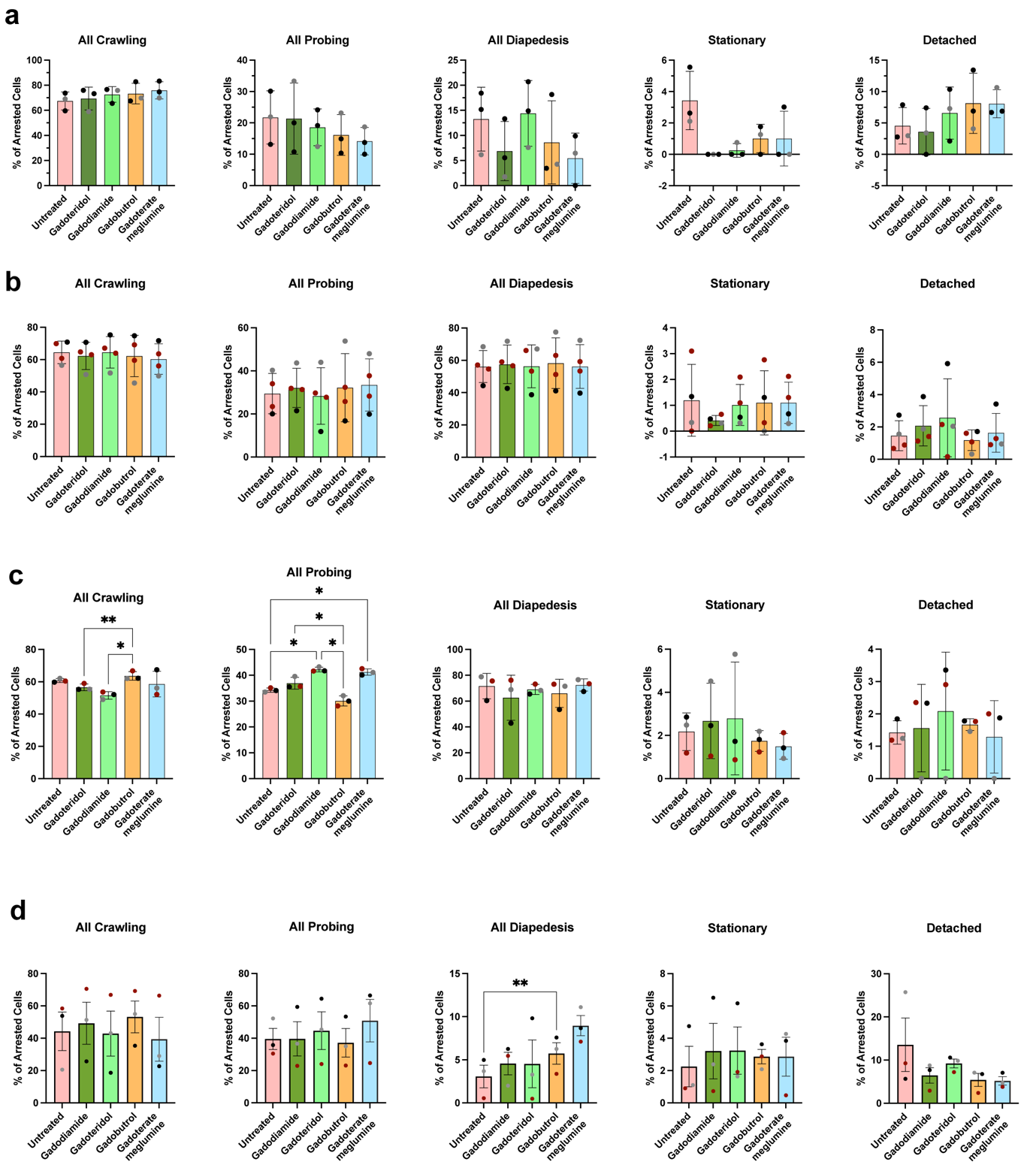
**

**Figure S6: GBCA treatment does not impair immune cell migration across the BBB under physiological flow *in vitro****.* PBMCs and monocytes were isolated from Buffy coat and CD4^+^ T cells and CD8^+^ T cells were *in vitro* activated by anti-CD3 and anti-CD28 polyclonal activation over 5 days. These cells at 1 million cells/mL were incubated directly at a 2 mM final GBCA concentration for 1 hour at 37°C, 5% CO_2_ before the experiment. Cells incubated with cell culture media only was used as control. Here, shown extended analysis of Fig. 4. Quantification of post-arrest behaviour of all arrested immune cells was quantified and categorised as crawling (all crawling), probing (all probing), performing diapedesis (all diapedesis), or stationary or detached for **(a)** PBMCs, **(b)** CD4^+^ T cells, **(c)** CD8^+^ T cells and **(d)** monocytes from atleast three independent experiments. The number of arrested cells for each condition was set to 100% and the behavioural categories shown as fraction thereof. Each dot is the mean value of 1-3 technical replicates within an experiment, and each colour represent values from a single donor performed as an independent experiment followed by Tukey’s multiple comparisons test. Data analysis was performed with one-way ANOVA with repeated measures per behavioural category followed by Tukey’s multiple comparisons test (p<0.05=*, p<0.01=**).

**Tables**
